# Correction: Copy number of pancreatic polypeptide receptor gene *NPY4R* correlates with body mass index and waist circumference

**DOI:** 10.1371/journal.pone.0276288

**Published:** 2022-10-12

**Authors:** Kateryna Shebanits, Johanna C. Andersson-Assarsson, Ingrid Larsson, Lena M. S. Carlsson, Lars Feuk, Dan Larhammar

## Notice of Republication

After this article [1] was published, it came to light that some individuals included in the study from the SOS SibPair panel were related. To address this, the authors performed a linear mixed model analysis that takes family relations into account, and provided an updated dataset and a revised version of the article that incorporates the new analysis and addresses reporting issues identified in the journal’s post-publication assessment.

The article was republished on October 4, 2022, to replace the original article with the updated, corrected version, and to remove a Supporting Information file (S1 Appendix) that was not needed to report the study or its findings. The Supporting Information dataset S1 Table was also replaced with the updated, corrected version. The originally published, uncorrected article and the republished, corrected article are provided here for reference.

The article text revisions include the following:

- In the Abstract, the sixth sentence was updated to: “Rather than the expected negative correlation, we observed a positive correlation between *NPY4R* copy number and BMI as well as waist circumference in women (r = 0.267, p = 2.65×10^−7^ and r = 0.256, p = 8×10^−7^, respectively).”
- In the Abstract, the p value in the sixth set of parentheses was corrected in the revised version to “(p = 2.8×10^−5^ and p = 6.2×10^−5^, respectively)”.
- In the Study populations subsection of the Materials and methods, a clarification of how the study sample was formed and a clarification of the BMI range of the study sample was added.
- In the Data analysis and statistics subsection of the Materials and methods, a description of the linear mixed model, an explanation of the analysis procedure, and the q-value used for the FDR have been added.
- Coefficient “r” was replaced with “Pearson’s r” in the Abstract and the Results section.
- In the Results section, in the third sentence of the second paragraph, “Linear Regression” was updated to “Linear Mixed model”.
- Several p values were updated in the Results section.
- In paragraph 4 of the Results section, the following sentence reported incorrect kcal/kg values: “Each additional copy was associated with a decrease of 3.09 kcal/kg in the whole study sample (p = 2×10−6) and a decrease of 3.54 kcal/kg in women separately (p = 8×10−6).” In the updated version, 3.09 kcal/kg was corrected to 3.07 kcal/kg, and 3.54 kcal/kg was corrected to 3.49 kcal/kg.
- In the Discussion section, the BMI range of included participants was reported incorrectly in the second paragraph of the section titled, “Inverse correlation between body weight, waist circumference and *NPY4R* copy number”. The correct BMI range is to 17–50 kg/m^2^, as is reported in the republished article.

The article’s main conclusions are not affected by the new analysis or article revisions.

A member of *PLOS ONE*’s Editorial Board reviewed the updates and advised that all issues were satisfactorily addressed in the republished version of the article.

## Supporting information

S1 File Originally published, uncorrected article. (PDF)

S2 File Republished, corrected article. (PDF)
