# Supplementary material for: Copy number of pancreatic polypeptide receptor gene NPY4R correlates with body mass index and waist circumference
Source: PLoS One. 2018 Apr 5;13(4):e0194668. doi: 10.1371/journal.pone.0194668 (PMC5886410; doi:10.1371/journal.pone.0194668)
Supplement: S1 Table — Full phenotype and NPY4R copy number data for 558 individuals used in this study. (DOCX) [file pone.0194668.s001.docx]

**S1 Table. Phenotype and genotype data.**

| **ID** | **Family ID** | **Age (years)** | **Sex** | **BMI**  **(kg/m^2^)** | **WC (cm)** | **NPY4R copy number** | **Total energy intake (kcal)** | **kcal/kg** |
| --- | --- | --- | --- | --- | --- | --- | --- | --- |
| A128 | 333 | 57 | 2 | 35 | 113 | 6,19 | 1920 | 21 |
| A155 | 59 | 44 | 2 | 44 | 123 | 4,00 | 2635 | 22 |
| A16 | 16 | 58 | 1 | 27 | 96 | 5,46 | 1697 | 20 |
| A164 | 55 | 37 | 1 | 35 | 116 | 3,80 | 4684 | 39 |
| A180 | 111 | 55 | 2 | 28 | 97 | 4,83 | 3401 | 41 |
| A218 | 68 | 42 | 2 | 42 | 116 | 5,42 | 2720 | 22 |
| A239 | 122 | 40 | 1 | 41 | 138 | 7,28 | 4747 | 31 |
| A275 | 147 | 53 | 2 | 26 | 90 | 4,23 | 2147 | 29 |
| A297 | 285 | 57 | 2 | 36 | 106 | 5,38 | 1080 | 12 |
| A384 | 212 | 56 | 2 | 41 | 120 | 4,24 | 2177 | 19 |
| A386 | 332 | 42 | 1 | 25 | 95 | 4,27 | 2919 | 34 |
| A390 | 22 | 68 | 2 | 30 | 105 | 4,58 | 1456 | 19 |
| A391 | 343 | 53 | 1 | 31 | 110 | 3,71 | 3516 | 35 |
| A396 | 50 | 24 | 2 | 30 | 101 | 3,92 | 2903 | 31 |
| A41 | 360 | 58 | 1 | 30 | 115 | 4,10 | 2083 | 19 |
| A413 | 70 | 39 | 2 | 42 | 122 | 4,79 | 1761 | 14 |
| A471 | 442 | 54 | 2 | 31 | 102 | 4,40 | 2148 | 26 |
| A515 | 364 | 55 | 1 | 33 | 109 | 4,31 | 2788 | 25 |
| A527 | 26 | 36 | 2 | 30 | 103 | 4,32 | 2215 | 26 |
| A550 | 151 | 36 | 2 | 45 | 125 | 5,23 | 2740 | 24 |
| A64 | 143 | 52 | 1 | 26 | 93 | 4,16 | 2910 | 31 |
| A83 | 25 | 63 | 1 | 27 | 92 | 4,27 | 2103 | 25 |
| B108 | 198 | 58 | 2 | 39 | 114 | 6,47 | 1815 | 16 |
| B133 | 181 | 57 | 1 | 43 | 125 | 4,94 | 2956 | 23 |
| B138 | 469 | 55 | 1 | 23 | 90 | 3,62 | 2880 | 38 |
| B161 | 20 | 70 | 1 | 30 | 113 | 3,34 | 2349 | 25 |
| B209 | 373 | 46 | 1 | 29 | 96 | 4,36 | 3807 | 45 |
| B240 | 185 | 38 | 2 | 37 | 117 | 3,20 | 3818 | 36 |
| B262 | 267 | 42 | 2 | 34 | 105 | 4,03 | 2411 | 25 |
| B306 | 115 | 63 | 2 | 29 | 95 | 4,36 | 1833 | 26 |
| B335 | 23 | 34 | 2 | 34 | 106 | 5,32 | 2230 | 23 |
| B350 | 366 | 49 | 1 | 31 | 110 | 4,13 | 3154 | 31 |
| B389 | 468 | 40 | 2 | 26 | 89 | 3,61 | 1450 | 18 |
| B42 | 16 | 56 | 2 | 27 | 90 | 4,35 | 2140 | 32 |
| B420 | 261 | 52 | 2 | 28 | 97 | 4,27 | 2019 | 27 |
| B434 | 357 | 48 | 1 | 26 | 91 | 4,23 | 4054 | 51 |
| B475 | 118 | 29 | 1 | 28 | 94 | 5,31 | 2645 | 28 |
| B477 | 40 | 40 | 1 | 32 | 111 | 4,50 | 3301 | 33 |
| B491 | 411 | 42 | 2 | 21 | 71 | 3,35 | 1468 | 25 |
| B528 | 296 | 45 | 2 | 19 | 64 | 4,17 | 1992 | 39 |
| B66 | 10 | 40 | 2 | 25 | 80 | 4,00 | 2354 | 36 |
| B7 | 66 | 39 | 2 | 42 | 121 | 5,22 | 1275 | 11 |
| B87 | 107 | 61 | 2 | 26 | 92 | 5,37 | 2134 | 31 |
| C109 | 133 | 44 | 2 | 27 | 85 | 3,99 | 2973 | 38 |
| C140 | 223 | 45 | 2 | 34 | 109 | 3,81 | 1340 | 13 |
| C148 | 353 | 48 | 1 | 26 | 82 | 3,77 | 3175 | 38 |
| C156 | 164 | 39 | 1 | 43 | 131 | 6,03 | 3424 | 24 |
| C175 | 418 | 54 | 2 | 36 | 108 | 4,24 | 1628 | 16 |
| C193 | 121 | 54 | 2 | 29 | 94 | 4,28 | 1609 | 19 |
| C21 | 266 | 59 | 1 | 32 | 110 | 3,91 | 2443 | 22 |
| C268 | 299 | 45 | 2 | 32 | 98 | 4,51 | 1525 | 18 |
| C301 | 207 | 44 | 2 | 42 | 121 | 4,35 | 3509 | 33 |
| C355 | 386 | 41 | 2 | 18 | 65 | 3,05 | 3759 | 79 |
| C383 | 124 | 56 | 1 | 32 | 109 | 7,51 | 1920 | 20 |
| C388 | 325 | 54 | 2 | 19 | 61 | 4,22 | 2842 | 65 |
| C457 | 113 | 31 | 2 | 35 | 117 | 4,91 | 2408 | 23 |
| C463 | 108 | 60 | 2 | 36 | 106 | 5,86 | 2086 | 21 |
| C464 | 199 | 51 | 1 | 35 | 119 | 5,46 | 2656 | 25 |
| C466 | 148 | 45 | 2 | 23 | 78 | 4,25 | 2225 | 34 |
| C50 | 262 | 40 | 1 | 33 | 115 | 3,14 | 4728 | 38 |
| C520 | 390 | 52 | 2 | 34 | 103 | 4,91 | 2551 | 32 |
| C556 | 432 | 58 | 1 | 31 | 105 | 4,30 | 3015 | 31 |
| C75 | 86 | 38 | 1 | 33 | 115 | 4,23 | 3004 | 26 |
| C79 | 35 | 39 | 2 | 35 | 108 | 4,49 | 2427 | 25 |
| D103 | 38 | 38 | 2 | 31 | 90 | 4,73 | 2016 | 22 |
| D111 | 88 | 34 | 2 | 44 | 125 | 4,58 | 2975 | 25 |
| D167 | 32 | 22 | 2 | 19 | 66 | 5,32 | 1298 | 21 |
| D173 | 12 | 76 | 1 | 25 | 90 | 4,45 | 3355 | 48 |
| D188 | 229 | 57 | 2 | 50 | 142 | 3,78 | 1796 | 12 |
| D227 | 151 | 38 | 2 | 23 | 72 | 4,20 | 2199 | 37 |
| D259 | 87 | 59 | 2 | 39 | 117 | 4,58 | 2465 | 24 |
| D26 | 168 | 57 | 2 | 33 | 101 | 4,61 | 1369 | 18 |
| D276 | 25 | 64 | 2 | 31 | 112 | 4,04 | 1925 | 22 |
| D277 | 196 | 39 | 2 | 41 | 118 | 4,13 | 3006 | 28 |
| D325 | 331 | 53 | 1 | 30 | 114 | 4,29 | 2439 | 26 |
| D329 | 423 | 51 | 2 | 24 | 80 | 4,81 | 2904 | 44 |
| D338 | 57 | 36 | 1 | 30 | 94 | 6,95 | 3469 | 36 |
| D406 | 270 | 59 | 1 | 26 | 93 | 4,62 | 2087 | 25 |
| D426 | 11 | 69 | 2 | 23 | 80 | 3,46 | 1391 | 24 |
| D44 | 438 | 43 | 2 | 33 | 103 | 4,52 | 2314 | 25 |
| D473 | 277 | 51 | 2 | 31 | 96 | 4,42 | 1956 | 22 |
| D511 | 21 | 39 | 1 | 26 | 87 | 4,37 | 2846 | 38 |
| D513 | 103 | 37 | 2 | 22 | 82 | 4,65 | 2990 | 51 |
| D523 | 124 | 54 | 2 | 26 | 83 | 5,53 | 1793 | 27 |
| D529 | 42 | 34 | 2 | 35 | 99 | 5,20 | 2269 | 22 |
| D537 | 47 | 42 | 2 | 41 | 116 | 4,53 | 1990 | 18 |
| D76 | 45 | 42 | 2 | 29 | 96 | 2,35 | 3105 | 38 |
| E118 | 195 | 57 | 2 | 42 | 112 | 4,28 | 2694 | 25 |
| E159 | 301 | 50 | 2 | 22 | 73 | 3,81 | 2993 | 46 |
| E174 | 297 | 59 | 2 | 37 | 100 | 4,44 | 1957 | 21 |
| E183 | 292 | 52 | 1 | 25 | 95 | 4,14 | 1748 | 25 |
| E187 | 224 | 52 | 2 | 41 | 122 | 3,13 | 4290 | 40 |
| E226 | 322 | 44 | 2 | 37 | 111 | 4,53 | 3411 | 35 |
| E270 | 457 | 56 | 2 | 40 | 112 | 4,36 | 4587 | 48 |
| E278 | 7 | 75 | 1 | 23 | 80 | 4,14 | 5437 | 82 |
| E284 | 218 | 55 | 2 | 47 | 131 | 4,23 | 1835 | 14 |
| E323 | 141 | 65 | 2 | 22 | 78 | 5,52 | 1486 | 27 |
| E36 | 422 | 38 | 2 | 21 | 71 | 3,59 | 1570 | 28 |
| E366 | 203 | 58 | 2 | 37 | 107 | 5,15 | 1488 | 15 |
| E392 | 342 | 46 | 1 | 27 | 90 | 4,69 | 3378 | 41 |
| E410 | 3 | 67 | 1 | 30 | 104 | 5,00 | 1595 | 19 |
| E442 | 82 | 36 | 1 | 30 | 100 | 5,25 | 2855 | 26 |
| E480 | 83 | 43 | 2 | 31 | 101 | 4,75 | 2622 | 31 |
| E519 | 182 | 57 | 1 | 39 | 150 | 4,18 | 1910 | 14 |
| E526 | 135 | 61 | 1 | 27 | 96 | 4,29 | 2970 | 32 |
| E71 | 114 | 52 | 1 | 35 | 108 | 5,14 | 2907 | 28 |
| E8 | 5 | 51 | 2 | 33 | 100 | 3,89 | 1674 | 19 |
| E85 | 4 | 69 | 1 | 25 | 84 | 4,11 | 3172 | 41 |
| F105 | 313 | 57 | 1 | 33 | 114 | 4,61 | 1766 | 17 |
| F13 | 133 | 48 | 1 | 27 | 96 | 6,34 | 3233 | 36 |
| F134 | 441 | 49 | 1 | 25 | 96 | 4,98 | 2476 | 27 |
| F177 | 314 | 57 | 1 | 29 | 96 | 4,64 | 1792 | 20 |
| F184 | 356 | 56 | 1 | 32 | 110 | 5,30 | 1801 | 18 |
| F228 | 35 | 56 | 2 | 25 | 88 | 4,51 | 1832 | 26 |
| F256 | 173 | 57 | 1 | 37 | 123 | 4,24 | 1630 | 13 |
| F27 | 132 | 47 | 2 | 32 | 99 | 4,91 | 2995 | 34 |
| F274 | 315 | 51 | 2 | 35 | 103 | 5,09 | 2388 | 27 |
| F320 | 263 | 37 | 1 | 31 | 109 | 3,81 | 5083 | 41 |
| F336 | 443 | 49 | 2 | 33 | 103 | 4,00 | 2083 | 21 |
| F352 | 375 | 49 | 1 | 31 | 110 | 4,39 | 3274 | 32 |
| F364 | 283 | 40 | 2 | 19 | 75 | 4,04 | 3030 | 49 |
| F427 | 85 | 42 | 2 | 36 | 120 | 4,91 | 2695 | 26 |
| F465 | 170 | 51 | 1 | 40 | 129 | 4,33 | 4315 | 34 |
| F478 | 117 | 63 | 2 | 25 | 91 | 4,40 | 1740 | 26 |
| F487 | 265 | 59 | 2 | 29 | 99 | 5,66 | 2867 | 38 |
| F495 | 369 | 60 | 1 | 31 | 105 | 4,38 | 2231 | 21 |
| F57 | 434 | 42 | 2 | 32 | 108 | 4,33 | 1476 | 17 |
| F97 | 34 | 38 | 2 | 17 | 69 | 5,12 | 3266 | 64 |
| G1 | 11 | 71 | 1 | 25 | 95 | 5,63 | 1582 | 20 |
| G125 | 112 | 57 | 1 | 26 | 88 | 5,37 | 2208 | 29 |
| G136 | 244 | 39 | 2 | 19 | 65 | 3,96 | 3213 | 65 |
| G146 | 73 | 33 | 2 | 39 | 121 | 5,00 | 2906 | 28 |
| G154 | 240 | 53 | 2 | 38 | 106 | 4,45 | 2784 | 30 |
| G185 | 220 | 44 | 2 | 42 | 129 | 5,21 | 1267 | 11 |
| G216 | 50 | 45 | 2 | 40 | 123 | 5,23 | 2228 | 19 |
| G298 | 36 | 36 | 2 | 34 | 105 | 5,20 | 2745 | 27 |
| G302 | 90 | 38 | 2 | 35 | 101 | 5,63 | 1295 | 14 |
| G309 | 83 | 72 | 2 | 33 | 111 | 6,86 | 2963 | 34 |
| G312 | 8 | 38 | 1 | 27 | 91 | 4,81 | 3251 | 40 |
| G361 | 351 | 58 | 1 | 25 | 99 | 4,33 | 4092 | 48 |
| G419 | 77 | 32 | 2 | 39 | 116 | 4,16 | 2992 | 26 |
| G432 | 114 | 52 | 2 | 27 | 86 | 4,32 | 3399 | 43 |
| G468 | 56 | 38 | 2 | 25 | 88 | 3,84 | 1300 | 17 |
| G482 | 319 | 51 | 2 | 31 | 116 | 3,77 | 1853 | 20 |
| G512 | 29 | 62 | 1 | 27 | 91 | 4,62 | 2683 | 33 |
| G52 | 379 | 51 | 1 | 28 | 105 | 4,13 | 3075 | 34 |
| G546 | 201 | 48 | 2 | 41 | 134 | 5,02 | 5500 | 45 |
| G60 | 190 | 39 | 2 | 41 | 117 | 4,24 | 1815 | 16 |
| G81 | 408 | 40 | 2 | 23 | 82 | 3,91 | 1648 | 25 |
| H101 | 389 | 40 | 2 | 22 | 79 | 3,83 | 2414 | 38 |
| H113 | 409 | 50 | 1 | 32 | 114 | 3,89 | 3114 | 34 |
| H12 | 59 | 50 | 1 | 34 | 109 | 5,30 | 4153 | 35 |
| H181 | 233 | 39 | 2 | 45 | 123 | 4,14 | 2800 | 25 |
| H194 | 344 | 50 | 2 | 19 | 71 | 4,88 | 2276 | 47 |
| H202 | 137 | 35 | 2 | 27 | 83 | 5,53 | 1026 | 16 |
| H231 | 242 | 57 | 2 | 20 | 68 | 3,74 | 3480 | 60 |
| H250 | 134 | 63 | 2 | 24 | 89 | 6,36 | 1337 | 21 |
| H279 | 246 | 38 | 2 | 32 | 96 | 2,83 | 1257 | 13 |
| H299 | 213 | 51 | 2 | 42 | 132 | 4,29 | 1731 | 14 |
| H342 | 71 | 45 | 2 | 44 | 106 | 4,69 | 1845 | 17 |
| H351 | 336 | 56 | 1 | 42 | 133 | 3,95 | 2136 | 15 |
| H360 | 230 | 56 | 2 | 40 | 119 | 5,56 | 3455 | 27 |
| H37 | 139 | 71 | 2 | 25 | 93 | 4,43 | 2490 | 34 |
| H438 | 1 | 64 | 2 | 37 | 119 | 4,21 | 1968 | 23 |
| H472 | 214 | 48 | 2 | 47 | 121 | 4,39 | 3834 | 30 |
| H485 | 99 | 34 | 2 | 41 | 119 | 4,96 | 3708 | 33 |
| H488 | 149 | 63 | 1 | 35 | 111 | 6,36 | 2074 | 21 |
| H506 | 67 | 71 | 1 | 30 | 108 | 3,58 | 1981 | 20 |
| H63 | 424 | 53 | 2 | 40 | 126 | 5,17 | 1330 | 11 |
| I107 | 200 | 53 | 2 | 45 | 136 | 4,39 | 2492 | 20 |
| I132 | 85 | 65 | 1 | 29 | 98 | 5,45 | 1344 | 15 |
| I139 | 228 | 45 | 2 | 37 | 114 | 4,63 | 2578 | 24 |
| I215 | 79 | 47 | 2 | 35 | 111 | 5,62 | 2581 | 26 |
| I22 | 217 | 46 | 2 | 47 | 130 | 4,03 | 2473 | 20 |
| I233 | 452 | 56 | 2 | 34 | 111 | 5,55 | 3303 | 34 |
| I235 | 329 | 56 | 2 | 25 | 85 | 3,34 | 6338 | 105 |
| I272 | 463 | 61 | 2 | 33 | 119 | 5,29 | 1822 | 18 |
| I293 | 147 | 61 | 1 | 35 | 109 | 4,50 | 3990 | 41 |
| I308 | 41 | 35 | 1 | 34 | 117 | 4,66 | 2726 | 24 |
| I348 | 149 | 61 | 2 | 33 | 113 | 3,81 | 2369 | 30 |
| I382 | 143 | 55 | 2 | 33 | 104 | 4,04 | 1874 | 24 |
| I416 | 339 | 59 | 1 | 40 | 123 | 3,97 | 2655 | 21 |
| I428 | 156 | 35 | 1 | 37 | 117 | 4,68 | 3638 | 28 |
| I444 | 59 | 33 | 1 | 29 | 97 | 4,33 | 4521 | 43 |
| I45 | 40 | 58 | 2 | 28 | 95 | 5,83 | 1343 | 17 |
| I459 | 295 | 38 | 1 | 31 | 103 | 5,23 | 2620 | 24 |
| I486 | 382 | 53 | 1 | 33 | 105 | 4,17 | 1911 | 20 |
| I535 | 125 | 43 | 1 | 26 | 91 | 4,47 | 2157 | 26 |
| I68 | 237 | 39 | 2 | 29 | 86 | 3,92 | 3969 | 52 |
| I86 | 335 | 56 | 2 | 23 | 83 | 3,69 | 2221 | 36 |
| J115 | 56 | 49 | 2 | 46 | 137 | 4,88 | 2226 | 17 |
| J15 | 72 | 37 | 2 | 38 | 108 | 4,75 | 2145 | 19 |
| J162 | 86 | 35 | 1 | 33 | 112 | 4,43 | 2156 | 18 |
| J182 | 136 | 49 | 1 | 30 | 107 | 4,79 | 2051 | 22 |
| J201 | 340 | 50 | 1 | 37 | 122 | 4,12 | 2522 | 18 |
| J220 | 74 | 57 | 2 | 27 | 94 | 5,59 | 3024 | 42 |
| J249 | 51 | 41 | 2 | 19 | 68 | 4,12 | 4797 | 90 |
| J286 | 436 | 56 | 1 | 32 | 111 | 5,53 | 2236 | 22 |
| J328 | 467 | 45 | 2 | 19 | 81 | 4,03 | 2414 | 41 |
| J341 | 31 | 49 | 1 | 31 | 106 | 5,01 | 1612 | 17 |
| J356 | 466 | 48 | 2 | 22 | 89 | 2,76 | 2569 | 40 |
| J357 | 273 | 51 | 1 | 26 | 100 | 3,67 | 3087 | 41 |
| J431 | 328 | 46 | 2 | 18 | 65 | 3,89 | 3101 | 63 |
| J436 | 308 | 45 | 1 | 26 | 94 | 4,03 | 1983 | 26 |
| J445 | 330 | 39 | 1 | 29 | 102 | 3,49 | 2632 | 26 |
| J452 | 276 | 54 | 2 | 19 | 76 | 4,15 | 2229 | 39 |
| J47 | 19 | 71 | 1 | 33 | 118 | 4,34 | 3060 | 34 |
| J516 | 41 | 59 | 2 | 28 | 88 | 4,39 | 1995 | 26 |
| J536 | 431 | 60 | 1 | 27 | 92 | 5,51 | 2745 | 35 |
| J549 | 294 | 49 | 2 | 34 | 106 | 4,64 | 1489 | 15 |
| J58 | 361 | 41 | 1 | 24 | 90 | 3,98 | 2862 | 34 |
| J88 | 352 | 52 | 1 | 32 | 114 | 3,96 | 1824 | 17 |
| K102 | 287 | 47 | 2 | 23 | 87 | 3,94 | 2304 | 34 |
| K122 | 52 | 39 | 2 | 38 | 116 | 3,85 | 2655 | 24 |
| K131 | 116 | 27 | 1 | 22 | 74 | 4,40 | 3374 | 52 |
| K171 | 413 | 53 | 1 | 26 | 93 | 5,52 | 1240 | 14 |
| K192 | 278 | 44 | 2 | 30 | 89 | 4,25 | 1600 | 20 |
| K197 | 140 | 56 | 1 | 30 | 105 | 4,56 | 2284 | 24 |
| K206 | 155 | 67 | 2 | 23 | 72 | 4,12 | 1564 | 26 |
| K25 | 72 | 35 | 2 | 36 | 107 | 4,71 | 2422 | 23 |
| K271 | 32 | 36 | 1 | 25 | 89 | 5,44 | 2877 | 37 |
| K285 | 388 | 48 | 2 | 23 | 79 | 4,07 | 1493 | 23 |
| K33 | 291 | 49 | 2 | 23 | 83 | 3,81 | 1167 | 17 |
| K333 | 65 | 41 | 2 | 39 | 120 | 4,05 | 2423 | 23 |
| K349 | 304 | 54 | 2 | 31 | 99 | 3,89 | 2453 | 30 |
| K375 | 79 | 78 | 1 | 30 | 106 | 4,02 | 4255 | 48 |
| K425 | 256 | 36 | 1 | 25 | 85 | 5,27 | 5545 | 72 |
| K469 | 415 | 38 | 1 | 26 | 93 | 3,92 | 3219 | 40 |
| K494 | 34 | 42 | 2 | 30 | 101 | 4,44 | 2628 | 29 |
| K503 | 17 | 68 | 1 | 23 | 87 | 4,17 | 2868 | 41 |
| K508 | 94 | 40 | 2 | 38 | 124 | 7,95 | 2226 | 19 |
| K521 | 68 | 52 | 2 | 31 | 95 | 6,64 | 1535 | 17 |
| K62 | 126 | 72 | 1 | 33 | 107 | 3,81 | 2795 | 29 |
| L116 | 64 | 44 | 2 | 40 | 104 | 4,59 | 1516 | 15 |
| L195 | 81 | 32 | 2 | 39 | 115 | 4,79 | 1888 | 18 |
| L2 | 2 | 79 | 1 | 25 | 96 | 5,51 | 2612 | 35 |
| L207 | 447 | 51 | 2 | 21 | 69 | 4,14 | 1956 | 29 |
| L245 | 268 | 58 | 2 | 33 | 109 | 4,10 | 2111 | 25 |
| L248 | 219 | 40 | 2 | 44 | 130 | 4,92 | 2204 | 16 |
| L255 | 163 | 39 | 2 | 40 | 115 | 3,41 | 2406 | 22 |
| L261 | 40 | 59 | 1 | 29 | 106 | 4,02 | 2932 | 33 |
| L281 | 27 | 34 | 2 | 31 | 96 | 3,26 | 2408 | 28 |
| L29 | 29 | 61 | 2 | 30 | 111 | 3,64 | 2341 | 28 |
| L332 | 36 | 58 | 2 | 25 | 96 | 4,37 | 2200 | 31 |
| L367 | 162 | 63 | 2 | 23 | 80 | 3,92 | 1518 | 25 |
| L370 | 395 | 41 | 1 | 29 | 96 | 4,31 | 4974 | 58 |
| L423 | 338 | 60 | 1 | 32 | 103 | 4,64 | 3891 | 43 |
| L439 | 324 | 58 | 2 | 35 | 105 | 4,02 | 1914 | 20 |
| L441 | 95 | 54 | 1 | 29 | 102 | 4,35 | 2397 | 26 |
| L474 | 248 | 47 | 1 | 30 | 110 | 4,47 | 2125 | 21 |
| L493 | 96 | 55 | 2 | 20 | 70 | 5,12 | 1653 | 29 |
| L530 | 309 | 55 | 2 | 28 | 83 | 6,62 | 2225 | 30 |
| L533 | 53 | 35 | 1 | 36 | 113 | 4,10 | 3455 | 30 |
| L554 | 459 | 61 | 1 | 27 | 94 | 4,45 | 2226 | 27 |
| L67 | 264 | 59 | 2 | 26 | 90 | 4,11 | 1922 | 25 |
| L99 | 359 | 55 | 1 | 34 | 114 | 4,90 | 1469 | 14 |
| M106 | 136 | 45 | 2 | 29 | 92 | 4,31 | 3590 | 46 |
| M14 | 51 | 46 | 1 | 27 | 96 | 4,97 | 2117 | 25 |
| M151 | 123 | 63 | 2 | 30 | 98 | 3,38 | 2809 | 35 |
| M196 | 323 | 60 | 2 | 31 | 110 | 4,33 | 1692 | 22 |
| M205 | 98 | 51 | 1 | 18 | 72 | 4,35 | 3194 | 55 |
| M208 | 215 | 51 | 2 | 46 | 134 | 6,26 | 2899 | 23 |
| M234 | 238 | 54 | 2 | 27 | 84 | 4,53 | 2519 | 39 |
| M282 | 57 | 38 | 2 | 34 | 106 | 5,07 | 1094 | 12 |
| M289 | 376 | 52 | 2 | 19 | 67 | 3,90 | 2152 | 44 |
| M294 | 269 | 46 | 2 | 29 | 90 | 4,96 | 2441 | 30 |
| M30 | 36 | 60 | 1 | 27 | 104 | 4,22 | 2175 | 24 |
| M316 | 371 | 61 | 1 | 30 | 106 | 4,30 | 3685 | 38 |
| M380 | 138 | 44 | 2 | 36 | 107 | 4,98 | 1280 | 13 |
| M403 | 281 | 51 | 2 | 27 | 84 | 4,21 | 1661 | 21 |
| M405 | 46 | 37 | 1 | 28 | 105 | 4,42 | 3470 | 35 |
| M462 | 439 | 61 | 1 | 30 | 110 | 4,71 | 2896 | 29 |
| M479 | 32 | 37 | 2 | 39 | 118 | 5,18 | 1532 | 13 |
| M504 | 305 | 39 | 2 | 23 | 79 | 3,59 | 1930 | 31 |
| M539 | 172 | 50 | 2 | 37 | 129 | 4,24 | 4309 | 44 |
| M551 | 349 | 37 | 2 | 19 | 77 | 4,61 | 2280 | 41 |
| M77 | 456 | 61 | 2 | 29 | 100 | 7,07 | 2711 | 31 |
| M95 | 192 | 48 | 1 | 44 | 150 | 3,92 | 3404 | 23 |
| N129 | 279 | 39 | 2 | 22 | 80 | 3,74 | 1878 | 31 |
| N150 | 258 | 47 | 2 | 20 | 75 | 3,66 | 4130 | 69 |
| N163 | 253 | 53 | 2 | 28 | 94 | 6,80 | 1810 | 25 |
| N166 | 398 | 55 | 2 | 23 | 80 | 4,10 | 1582 | 23 |
| N18 | 95 | 19 | 1 | 50 | 138 | 4,71 | 4948 | 31 |
| N189 | 365 | 59 | 1 | 34 | 113 | 4,31 | 5778 | 50 |
| N257 | 24 | 37 | 2 | 35 | 100 | 4,16 | 2594 | 28 |
| N346 | 412 | 50 | 1 | 27 | 93 | 4,21 | 3681 | 47 |
| N354 | 15 | 44 | 2 | 42 | 113 | 4,51 | 3161 | 32 |
| N376 | 191 | 45 | 2 | 38 | 125 | 5,39 | 2582 | 24 |
| N377 | 150 | 72 | 1 | 31 | 109 | 4,51 | 3260 | 34 |
| N379 | 450 | 60 | 2 | 28 | 99 | 4,00 | 2652 | 36 |
| N400 | 43 | 71 | 2 | 34 | 107 | 4,09 | 2717 | 31 |
| N433 | 429 | 53 | 1 | 18 | 72 | 4,20 | 2458 | 46 |
| N446 | 282 | 48 | 2 | 25 | 87 | 4,77 | 2402 | 37 |
| N458 | 188 | 44 | 2 | 35 | 107 | 4,87 | 1716 | 17 |
| N46 | 46 | 33 | 2 | 33 | 115 | 4,04 | 3233 | 32 |
| N460 | 70 | 67 | 2 | 25 | 89 | 4,58 | 1543 | 22 |
| N543 | 249 | 46 | 1 | 25 | 96 | 4,22 | 2854 | 32 |
| N547 | 69 | 49 | 2 | 38 | 124 | 4,50 | 1400 | 12 |
| N55 | 27 | 66 | 2 | 28 | 88 | 4,28 | 2472 | 30 |
| N93 | 465 | 58 | 2 | 31 | 111 | 4,42 | 3063 | 38 |
| O123 | 167 | 50 | 1 | 33 | 111 | 4,22 | 3012 | 28 |
| O137 | 126 | 71 | 2 | 41 | 131 | 5,12 | 3580 | 34 |
| O176 | 397 | 50 | 1 | 37 | 126 | 3,03 | 2228 | 20 |
| O204 | 472 | 48 | 2 | 18 | 64 | 3,84 | 2520 | 50 |
| O23 | 458 | 49 | 2 | 26 | 89 | 5,48 | 2263 | 32 |
| O241 | 55 | 45 | 1 | 28 | 98 | 4,51 | 4359 | 47 |
| O258 | 140 | 54 | 2 | 25 | 84 | 5,14 | 1615 | 25 |
| O300 | 116 | 35 | 2 | 33 | 101 | 5,19 | 3336 | 35 |
| O326 | 473 | 60 | 1 | 29 | 106 | 5,14 | 1979 | 19 |
| O327 | 254 | 39 | 2 | 24 | 86 | 3,78 | 2205 | 35 |
| O330 | 227 | 57 | 2 | 39 | 117 | 4,54 | 2556 | 24 |
| O343 | 307 | 53 | 2 | 23 | 76 | 2,74 | 1250 | 23 |
| O397 | 120 | 32 | 2 | 36 | 109 | 5,45 | 2134 | 21 |
| O404 | 57 | 59 | 2 | 28 | 94 | 4,39 | 2541 | 37 |
| O430 | 180 | 53 | 2 | 40 | 122 | 4,19 | 2503 | 25 |
| O483 | 399 | 39 | 2 | 22 | 72 | 3,56 | 2783 | 42 |
| O49 | 176 | 58 | 2 | 34 | 113 | 5,36 | 1545 | 16 |
| O507 | 34 | 36 | 2 | 43 | 119 | 5,49 | 2738 | 21 |
| O541 | 23 | 58 | 1 | 29 | 99 | 4,48 | 2667 | 32 |
| O72 | 393 | 49 | 2 | 24 | 76 | 3,98 | 1890 | 32 |
| O80 | 174 | 50 | 2 | 39 | 119 | 5,73 | 1840 | 17 |
| P120 | 73 | 51 | 1 | 29 | 100 | 4,84 | 1879 | 21 |
| P19 | 6 | 42 | 2 | 38 | 109 | 5,11 | 3972 | 42 |
| P191 | 272 | 58 | 2 | 34 | 101 | 3,88 | 2460 | 27 |
| P199 | 449 | 45 | 1 | 29 | 99 | 4,32 | 2700 | 32 |
| P211 | 39 | 37 | 2 | 33 | 111 | 4,33 | 1719 | 19 |
| P237 | 28 | 41 | 2 | 36 | 111 | 4,28 | 2876 | 26 |
| P247 | 54 | 44 | 2 | 26 | 92 | 3,21 | 1640 | 21 |
| P263 | 311 | 43 | 2 | 38 | 112 | 5,15 | 3225 | 30 |
| P264 | 462 | 42 | 2 | 26 | 91 | 4,20 | 2101 | 28 |
| P291 | 428 | 61 | 1 | 30 | 104 | 4,45 | 2975 | 31 |
| P305 | 221 | 40 | 2 | 43 | 107 | 4,68 | 2753 | 24 |
| P314 | 69 | 46 | 2 | 29 | 99 | 4,05 | 1604 | 17 |
| P417 | 378 | 42 | 1 | 28 | 92 | 2,10 | 2242 | 27 |
| P43 | 17 | 67 | 2 | 25 | 83 | 4,06 | 2429 | 41 |
| P447 | 321 | 39 | 2 | 44 | 131 | 4,90 | 3110 | 24 |
| P454 | 189 | 50 | 2 | 46 | 117 | 4,17 | 1200 | 12 |
| P492 | 446 | 42 | 1 | 28 | 92 | 4,52 | 2011 | 26 |
| P518 | 232 | 51 | 2 | 37 | 109 | 4,20 | 2076 | 19 |
| P59 | 37 | 47 | 1 | 32 | 113 | 2,90 | 1905 | 18 |
| P94 | 33 | 44 | 2 | 29 | 102 | 4,45 | 1480 | 19 |
| Q112 | 158 | 58 | 1 | 28 | 99 | 4,19 | 2455 | 28 |
| Q203 | 63 | 42 | 2 | 43 | 116 | 7,49 | 2078 | 17 |
| Q210 | 370 | 61 | 1 | 33 | 105 | 4,64 | 3546 | 34 |
| Q222 | 135 | 57 | 2 | 31 | 98 | 4,95 | 1630 | 18 |
| Q224 | 392 | 38 | 1 | 28 | 93 | 4,40 | 4639 | 51 |
| Q229 | 383 | 52 | 2 | 26 | 90 | 6,50 | 1759 | 22 |
| Q288 | 91 | 48 | 2 | 38 | 111 | 4,06 | 3505 | 39 |
| Q321 | 286 | 42 | 2 | 21 | 80 | 2,92 | 3541 | 56 |
| Q331 | 102 | 47 | 2 | 27 | 95 | 4,50 | 2257 | 29 |
| Q372 | 247 | 56 | 2 | 31 | 94 | 4,08 | 2690 | 32 |
| Q374 | 396 | 53 | 1 | 25 | 98 | 4,93 | 2039 | 24 |
| Q393 | 111 | 58 | 1 | 25 | 87 | 5,18 | 2493 | 33 |
| Q407 | 384 | 41 | 2 | 23 | 75 | 3,74 | 2456 | 42 |
| Q412 | 22 | 70 | 1 | 31 | 110 | 4,99 | 2488 | 27 |
| Q415 | 55 | 39 | 1 | 25 | 89 | 4,51 | 2671 | 31 |
| Q440 | 451 | 50 | 2 | 24 | 93 | 3,88 | 2702 | 43 |
| Q5 | 290 | 52 | 2 | 23 | 70 | 3,65 | 2439 | 39 |
| Q51 | 274 | 42 | 2 | 45 | 122 | 4,09 | 4938 | 37 |
| Q542 | 454 | 50 | 1 | 25 | 101 | 4,43 | 3574 | 41 |
| Q557 | 132 | 49 | 1 | 27 | 93 | 4,56 | 4187 | 47 |
| Q56 | 146 | 45 | 1 | 37 | 117 | 4,38 | 1803 | 16 |
| Q92 | 38 | 36 | 1 | 29 | 99 | 4,09 | 4071 | 41 |
| R127 | 461 | 43 | 1 | 38 | 118 | 5,36 | 1885 | 14 |
| R157 | 205 | 57 | 2 | 41 | 120 | 3,98 | 2727 | 25 |
| R170 | 183 | 40 | 2 | 44 | 126 | 4,20 | 3303 | 27 |
| R178 | 433 | 57 | 1 | 29 | 98 | 5,91 | 3131 | 33 |
| R214 | 252 | 55 | 2 | 28 | 90 | 4,90 | 2089 | 25 |
| R217 | 68 | 51 | 1 | 26 | 94 | 6,62 | 3250 | 34 |
| R307 | 448 | 59 | 1 | 24 | 94 | 3,59 | 2724 | 36 |
| R337 | 60 | 39 | 1 | 25 | 90 | 4,54 | 2413 | 26 |
| R34 | 391 | 54 | 1 | 26 | 88 | 4,10 | 3044 | 39 |
| R345 | 127 | 39 | 1 | 36 | 117 | 6,21 | 7657 | 63 |
| R358 | 82 | 39 | 2 | 42 | 123 | 5,78 | 2854 | 25 |
| R373 | 417 | 49 | 1 | 23 | 83 | 3,95 | 3784 | 50 |
| R402 | 206 | 55 | 2 | 41 | 122 | 4,29 | 1772 | 17 |
| R456 | 175 | 46 | 2 | 40 | 108 | 5,71 | 3844 | 39 |
| R467 | 234 | 39 | 2 | 39 | 120 | 4,66 | 2631 | 26 |
| R509 | 310 | 45 | 2 | 34 | 113 | 4,56 | 2568 | 29 |
| R517 | 403 | 56 | 2 | 19 | 66 | 3,83 | 1735 | 39 |
| R53 | 45 | 37 | 2 | 19 | 66 | 5,32 | 2109 | 43 |
| R531 | 470 | 54 | 1 | 31 | 105 | 3,61 | 2303 | 22 |
| R538 | 407 | 46 | 1 | 34 | 110 | 4,38 | 4350 | 42 |
| R545 | 260 | 59 | 2 | 27 | 81 | 4,25 | 2364 | 38 |
| R89 | 255 | 53 | 2 | 23 | 77 | 3,69 | 1133 | 17 |
| R9 | 362 | 37 | 1 | 28 | 98 | 4,14 | 3565 | 39 |
| S124 | 101 | 34 | 1 | 44 | 134 | 3,81 | 2583 | 20 |
| S169 | 14 | 37 | 2 | 33 | 113 | 4,61 | 2064 | 20 |
| S17 | 271 | 49 | 2 | 29 | 95 | 4,09 | 1673 | 21 |
| S172 | 284 | 48 | 2 | 22 | 79 | 3,53 | 2698 | 42 |
| S179 | 406 | 41 | 2 | 24 | 82 | 3,74 | 1850 | 28 |
| S230 | 337 | 56 | 1 | 42 | 133 | 3,95 | 2136 | 15 |
| S232 | 416 | 49 | 2 | 19 | 70 | 4,26 | 3304 | 56 |
| S280 | 86 | 28 | 2 | 18 | 65 | 3,96 | 1559 | 32 |
| S295 | 148 | 47 | 1 | 26 | 84 | 4,56 | 1719 | 21 |
| S32 | 158 | 57 | 2 | 27 | 92 | 4,28 | 1668 | 23 |
| S322 | 54 | 37 | 2 | 35 | 107 | 3,99 | 2744 | 28 |
| S324 | 112 | 53 | 2 | 24 | 87 | 4,37 | 2131 | 34 |
| S387 | 300 | 46 | 1 | 33 | 99 | 4,29 | 1723 | 21 |
| S398 | 169 | 56 | 2 | 40 | 116 | 4,39 | 2616 | 27 |
| S421 | 358 | 43 | 1 | 25 | 94 | 5,24 | 2286 | 33 |
| S470 | 165 | 55 | 2 | 39 | 102 | 4,25 | 1813 | 19 |
| S481 | 440 | 43 | 1 | 29 | 105 | 5,26 | 1456 | 14 |
| S497 | 110 | 38 | 2 | 36 | 106 | 3,86 | 1214 | 12 |
| S524 | 317 | 40 | 2 | 33 | 99 | 4,71 | 1644 | 18 |
| S525 | 166 | 51 | 1 | 37 | 132 | 5,60 | 2855 | 22 |
| S54 | 211 | 50 | 2 | 42 | 138 | 4,17 | 2220 | 20 |
| S540 | 303 | 42 | 1 | 22 | 87 | 3,67 | 2748 | 35 |
| S552 | 245 | 57 | 2 | 30 | 98 | 3,17 | 2826 | 36 |
| S84 | 142 | 37 | 2 | 35 | 109 | 5,10 | 3108 | 33 |
| T119 | 368 | 53 | 1 | 33 | 101 | 5,56 | 1445 | 14 |
| T135 | 128 | 37 | 2 | 38 | 119 | 5,52 | 3859 | 37 |
| T147 | 179 | 54 | 1 | 39 | 124 | 4,19 | 1972 | 17 |
| T190 | 250 | 60 | 1 | 34 | 117 | 4,56 | 2691 | 26 |
| T236 | 455 | 39 | 1 | 32 | 107 | 6,05 | 2357 | 23 |
| T243 | 109 | 53 | 2 | 26 | 88 | 4,50 | 2086 | 27 |
| T315 | 48 | 35 | 2 | 40 | 116 | 4,50 | 2245 | 20 |
| T318 | 377 | 57 | 1 | 33 | 114 | 6,42 | 3505 | 31 |
| T319 | 160 | 50 | 2 | 26 | 94 | 4,75 | 3316 | 45 |
| T35 | 58 | 43 | 2 | 42 | 119 | 3,82 | 2611 | 22 |
| T359 | 216 | 40 | 2 | 46 | 117 | 5,55 | 1147 | 9 |
| T362 | 117 | 63 | 1 | 30 | 105 | 5,17 | 2298 | 24 |
| T414 | 453 | 55 | 2 | 20 | 70 | 4,51 | 1415 | 25 |
| T435 | 257 | 55 | 2 | 23 | 92 | 3,70 | 2133 | 29 |
| T453 | 52 | 30 | 2 | 31 | 99 | 4,82 | 2721 | 30 |
| T461 | 109 | 52 | 1 | 28 | 93 | 5,00 | 2752 | 31 |
| T490 | 437 | 53 | 2 | 39 | 111 | 4,47 | 1181 | 10 |
| T534 | 58 | 43 | 2 | 25 | 79 | 5,04 | 1884 | 27 |
| T6 | 105 | 25 | 1 | 26 | 88 | 5,76 | 1985 | 23 |
| T65 | 348 | 58 | 1 | 32 | 107 | 4,22 | 3252 | 32 |
| T98 | 210 | 48 | 2 | 39 | 115 | 4,27 | 2112 | 19 |
| U10 | 236 | 50 | 2 | 43 | 121 | 4,20 | 5112 | 43 |
| U104 | 194 | 53 | 2 | 41 | 113 | 5,41 | 2519 | 24 |
| U130 | 385 | 56 | 1 | 19 | 69 | 4,99 | 3098 | 53 |
| U149 | 306 | 38 | 2 | 21 | 72 | 3,70 | 2154 | 37 |
| U158 | 64 | 68 | 1 | 29 | 97 | 4,66 | 2049 | 24 |
| U165 | 2 | 55 | 2 | 46 | 134 | 6,46 | 2008 | 15 |
| U168 | 26 | 71 | 1 | 25 | 95 | 4,81 | 2185 | 30 |
| U200 | 243 | 60 | 2 | 32 | 110 | 3,00 | 1936 | 22 |
| U266 | 239 | 49 | 2 | 32 | 95 | 4,58 | 2953 | 34 |
| U28 | 209 | 40 | 2 | 36 | 94 | 4,47 | 1829 | 19 |
| U283 | 355 | 37 | 2 | 24 | 86 | 4,98 | 2057 | 27 |
| U296 | 74 | 33 | 1 | 35 | 117 | 4,62 | 5490 | 48 |
| U313 | 341 | 49 | 1 | 31 | 107 | 4,06 | 2747 | 25 |
| U317 | 144 | 36 | 1 | 28 | 102 | 4,05 | 2833 | 27 |
| U401 | 131 | 47 | 2 | 38 | 117 | 4,20 | 2143 | 22 |
| U411 | 474 | 55 | 1 | 30 | 101 | 4,45 | 2774 | 28 |
| U424 | 102 | 51 | 1 | 35 | 116 | 5,22 | 2393 | 21 |
| U450 | 426 | 59 | 2 | 28 | 82 | 4,62 | 2410 | 36 |
| U498 | 380 | 50 | 2 | 36 | 107 | 5,73 | 2531 | 26 |
| U522 | 350 | 61 | 1 | 26 | 95 | 4,57 | 3258 | 44 |
| U532 | 312 | 44 | 2 | 21 | 81 | 4,04 | 2249 | 41 |
| U69 | 160 | 50 | 1 | 27 | 101 | 4,68 | 3071 | 31 |
| V121 | 96 | 56 | 1 | 34 | 114 | 5,22 | 3137 | 30 |
| V141 | 235 | 45 | 2 | 43 | 119 | 4,42 | 3421 | 28 |
| V142 | 13 | 52 | 2 | 43 | 135 | 3,94 | 1618 | 14 |
| V152 | 157 | 18 | 1 | 18 | 74 | 3,20 | 6109 | 92 |
| V238 | 471 | 43 | 1 | 19 | 74 | 4,62 | 4325 | 65 |
| V254 | 177 | 60 | 2 | 43 | 134 | 5,07 | 2842 | 24 |
| V265 | 5 | 54 | 2 | 41 | 112 | 5,22 | 1676 | 13 |
| V3 | 419 | 56 | 1 | 25 | 86 | 5,50 | 3008 | 39 |
| V31 | 49 | 38 | 2 | 31 | 96 | 4,40 | 1883 | 19 |
| V311 | 202 | 44 | 2 | 41 | 113 | 4,43 | 2986 | 28 |
| V340 | 372 | 49 | 1 | 30 | 104 | 4,14 | 3354 | 33 |
| V363 | 401 | 58 | 1 | 18 | 72 | 4,21 | 2561 | 48 |
| V378 | 84 | 57 | 2 | 29 | 98 | 4,73 | 2860 | 35 |
| V394 | 241 | 57 | 2 | 29 | 96 | 4,21 | 1561 | 20 |
| V418 | 171 | 55 | 2 | 40 | 106 | 6,23 | 2802 | 28 |
| V443 | 394 | 37 | 1 | 25 | 86 | 4,14 | 3829 | 48 |
| V448 | 149 | 39 | 1 | 27 | 91 | 4,39 | 3841 | 48 |
| V500 | 400 | 55 | 2 | 32 | 103 | 4,18 | 1486 | 16 |
| V558 | 106 | 69 | 2 | 33 | 105 | 3,48 | 2657 | 29 |
| V74 | 460 | 47 | 1 | 25 | 89 | 4,12 | 3487 | 42 |
| V91 | 289 | 57 | 2 | 33 | 96 | 5,15 | 1612 | 19 |
| W11 | 259 | 46 | 2 | 30 | 103 | 4,28 | 2374 | 27 |
| W110 | 298 | 57 | 2 | 26 | 72 | 4,61 | 1829 | 30 |
| W153 | 53 | 65 | 2 | 29 | 92 | 3,25 | 1754 | 22 |
| W160 | 354 | 39 | 1 | 23 | 84 | 2,85 | 2064 | 28 |
| W221 | 421 | 40 | 1 | 19 | 78 | 4,79 | 2572 | 43 |
| W223 | 100 | 43 | 1 | 29 | 103 | 4,99 | 4464 | 45 |
| W252 | 316 | 51 | 2 | 35 | 103 | 5,09 | 2388 | 27 |
| W267 | 251 | 60 | 2 | 32 | 102 | 4,03 | 2083 | 25 |
| W310 | 68 | 45 | 2 | 30 | 101 | 6,23 | 2113 | 23 |
| W334 | 280 | 37 | 2 | 24 | 80 | 3,90 | 2505 | 37 |
| W371 | 445 | 51 | 1 | 25 | 90 | 3,69 | 2717 | 36 |
| W38 | 222 | 50 | 2 | 40 | 112 | 6,58 | 2822 | 30 |
| W385 | 123 | 67 | 1 | 32 | 102 | 4,84 | 3086 | 34 |
| W408 | 69 | 53 | 1 | 30 | 100 | 4,75 | 3365 | 32 |
| W451 | 184 | 52 | 2 | 43 | 126 | 4,12 | 2716 | 25 |
| W455 | 275 | 38 | 1 | 27 | 92 | 5,87 | 2698 | 33 |
| W510 | 420 | 49 | 1 | 41 | 129 | 3,82 | 3474 | 26 |
| W514 | 187 | 48 | 2 | 42 | 116 | 6,16 | 3933 | 34 |
| W548 | 104 | 42 | 2 | 34 | 101 | 3,92 | 2486 | 29 |
| W555 | 334 | 44 | 2 | 32 | 101 | 5,29 | 2824 | 31 |
| W73 | 231 | 40 | 2 | 44 | 97 | 3,84 | 1865 | 16 |
| W82 | 363 | 54 | 1 | 37 | 125 | 4,82 | 3402 | 26 |
| X114 | 427 | 52 | 2 | 19 | 69 | 3,74 | 2343 | 47 |
| X144 | 402 | 54 | 1 | 30 | 107 | 5,13 | 1935 | 20 |
| X145 | 150 | 66 | 2 | 29 | 99 | 4,62 | 2089 | 29 |
| X20 | 65 | 34 | 1 | 29 | 99 | 4,68 | 1460 | 17 |
| X213 | 7 | 50 | 2 | 46 | 130 | 5,20 | 1487 | 12 |
| X225 | 425 | 59 | 2 | 33 | 107 | 4,69 | 2453 | 28 |
| X242 | 92 | 45 | 2 | 37 | 118 | 4,27 | 2635 | 24 |
| X269 | 226 | 39 | 2 | 47 | 132 | 4,39 | 2038 | 16 |
| X273 | 293 | 50 | 2 | 25 | 86 | 3,72 | 1904 | 27 |
| X287 | 302 | 58 | 1 | 32 | 100 | 3,93 | 3042 | 31 |
| X347 | 475 | 45 | 1 | 19 | 74 | 5,12 | 2392 | 35 |
| X368 | 405 | 43 | 2 | 20 | 68 | 3,95 | 1972 | 39 |
| X409 | 318 | 54 | 2 | 27 | 86 | 4,58 | 1979 | 28 |
| X449 | 30 | 38 | 2 | 35 | 117 | 3,96 | 2034 | 20 |
| X476 | 464 | 57 | 1 | 30 | 101 | 4,47 | 1470 | 15 |
| X48 | 134 | 62 | 1 | 25 | 87 | 4,41 | 2215 | 30 |
| X499 | 346 | 43 | 1 | 28 | 94 | 4,35 | 2148 | 25 |
| X505 | 387 | 38 | 2 | 24 | 78 | 4,78 | 1943 | 26 |
| X544 | 197 | 44 | 2 | 40 | 116 | 5,22 | 2247 | 19 |
| X78 | 137 | 69 | 2 | 32 | 106 | 7,10 | 1868 | 23 |
| X96 | 193 | 53 | 2 | 42 | 119 | 4,14 | 2038 | 17 |
| Y126 | 121 | 54 | 1 | 34 | 119 | 4,90 | 1811 | 16 |
| Y143 | 404 | 51 | 2 | 30 | 89 | 7,75 | 1584 | 20 |
| Y212 | 225 | 38 | 2 | 45 | 116 | 4,45 | 2637 | 24 |
| Y219 | 327 | 49 | 2 | 33 | 101 | 4,80 | 2714 | 27 |
| Y244 | 109 | 28 | 2 | 19 | 68 | 4,17 | 2072 | 37 |
| Y246 | 162 | 67 | 1 | 29 | 102 | 3,75 | 3682 | 40 |
| Y292 | 119 | 41 | 2 | 39 | 113 | 4,51 | 1382 | 14 |
| Y339 | 430 | 39 | 1 | 19 | 78 | 3,82 | 3458 | 57 |
| Y344 | 326 | 51 | 1 | 33 | 111 | 3,06 | 2812 | 26 |
| Y353 | 66 | 71 | 2 | 39 | 115 | 4,08 | 2243 | 22 |
| Y381 | 153 | 65 | 2 | 29 | 98 | 4,07 | 2064 | 24 |
| Y39 | 18 | 59 | 2 | 29 | 88 | 4,34 | 2029 | 30 |
| Y399 | 367 | 55 | 1 | 28 | 101 | 5,05 | 1283 | 14 |
| Y4 | 444 | 46 | 2 | 25 | 85 | 4,16 | 1505 | 23 |
| Y422 | 129 | 43 | 2 | 26 | 93 | 5,28 | 1654 | 22 |
| Y437 | 414 | 48 | 1 | 29 | 96 | 4,07 | 4089 | 48 |
| Y489 | 186 | 52 | 1 | 45 | 136 | 6,47 | 3542 | 24 |
| Y496 | 145 | 41 | 2 | 27 | 99 | 4,31 | 5189 | 64 |
| Y70 | 44 | 48 | 2 | 38 | 121 | 4,11 | 4615 | 36 |
| Y90 | 9 | 74 | 1 | 25 | 90 | 7,06 | 2764 | 35 |
| Z100 | 84 | 37 | 2 | 41 | 137 | 4,96 | 2815 | 24 |
| Z117 | 130 | 37 | 2 | 37 | 109 | 5,40 | 4259 | 41 |
| Z186 | 98 | 53 | 2 | 21 | 73 | 4,09 | 2527 | 40 |
| Z198 | 75 | 41 | 2 | 37 | 108 | 4,23 | 2765 | 25 |
| Z24 | 345 | 61 | 1 | 25 | 94 | 4,31 | 2946 | 34 |
| Z251 | 320 | 48 | 1 | 30 | 112 | 6,80 | 1613 | 15 |
| Z253 | 347 | 39 | 1 | 20 | 76 | 4,28 | 1973 | 31 |
| Z260 | 19 | 63 | 2 | 30 | 103 | 4,83 | 2118 | 25 |
| Z290 | 204 | 38 | 1 | 49 | 151 | 4,06 | 6270 | 35 |
| Z303 | 374 | 55 | 1 | 28 | 107 | 4,38 | 2789 | 30 |
| Z304 | 288 | 41 | 2 | 21 | 71 | 3,94 | 1075 | 18 |
| Z365 | 161 | 72 | 1 | 30 | 114 | 6,39 | 3227 | 33 |
| Z369 | 381 | 48 | 2 | 30 | 88 | 4,17 | 1743 | 24 |
| Z395 | 1 | 36 | 1 | 26 | 89 | 4,30 | 4312 | 55 |
| Z40 | 20 | 67 | 2 | 30 | 100 | 4,30 | 2603 | 35 |
| Z429 | 178 | 42 | 2 | 45 | 111 | 4,68 | 1926 | 17 |
| Z484 | 43 | 71 | 1 | 25 | 84 | 4,49 | 3325 | 45 |
| Z501 | 159 | 40 | 1 | 19 | 89 | 3,89 | 4447 | 77 |
| Z502 | 435 | 61 | 1 | 32 | 115 | 4,57 | 3117 | 28 |
| Z553 | 208 | 53 | 2 | 40 | 118 | 5,49 | 2269 | 19 |
| Z61 | 410 | 37 | 1 | 28 | 113 | 3,89 | 2158 | 22 |

Phenotype data, *NPY4R* copy number, self-reported total energy intake and total energy intake adjusted for body weight for 558 individuals used in this study. Sex is encoded as 1 for men and 2 for women.
