# Supplementary material for: Copy number of pancreatic polypeptide receptor gene NPY4R correlates with body mass index and waist circumference
Source: PLoS One. 2018 Apr 5;13(4):e0194668. doi: 10.1371/journal.pone.0194668 (PMC5886410; doi:10.1371/journal.pone.0194668)
Supplement: S2 Table — (DOCX) [file pone.0194668.s002.docx]

| Sample ID | Population | *NPY4R*  Copy number |
| --- | --- | --- |
| NA10847 | CEU | 4.1 |
| NA10851 | CEU | 6.2 |
| NA12155 | CEU | 5.6 |
| NA12717 | CEU | 3.9 |
| NA12878 | CEU | 3.0 |
| NA18524 | CHB | 4.1 |
| NA18529 | CHB | 4.3 |
| NA18536 | CHB | 4.6 |
| NA18542 | CHB | 3.9 |
| NA18603 | CHB | 4.5 |
| NA18627 | CHB | 4.2 |
| NA18745 | CHB | 4.4 |
| NA18760 | CHB | 3.6 |
| NA18795 | CHB | 4.0 |

**S2 Table. *NPY4R* copy number determined by ddPCR in Chinese and Caucasian samples from 1000 Genomes Project**

*NPY4R* copy number was measured by ddPCR using the protocol described in the Materials and methods section.
