# Supplementary material for: Correction: Copy number of pancreatic polypeptide receptor gene NPY4R correlates with body mass index and waist circumference
Source: PLoS One. 2022 Oct 12;17(10):e0276288. doi: 10.1371/journal.pone.0276288 (PMC9555669; doi:10.1371/journal.pone.0276288)
Supplement: S2 File — (PDF) [file pone.0276288.s002.pdf]

PLOS ONE

RESEARCH ARTICLE

Copy number of pancreatic polypeptide receptor gene *NPY4R* correlates with body mass index and waist circumference

Kateryna Shebanits[1], Johanna C. Andersson-Assarsson[2], Ingrid Larsson[3], Lena M. S. Carlsson[2], Lars Feuk[4], Dan Larhammar[1]*

1 Dept. of Neuroscience, Uppsala University, Uppsala, Sweden, **2** Dept. of Molecular and Clinical Medicine, Sahlgrenska Academy at Gothenburg University, Gothenburg, Sweden, **3** Dept. of Gastroenterology and Hepatology, Sahlgrenska University Hospital, Gothenburg, Sweden, **4** Dept. of Immunology, Genetics and Pathology, Science for Life Laboratory, Uppsala University, Uppsala, Sweden

* dan.larhammar@neuro.uu.se

Check for updates

Abstract

Multiple genetic studies have linked copy number variation (CNV) in different genes to body mass index (BMI) and obesity. A CNV on chromosome 10q11.22 has been associated with body weight. This CNV region spans *NPY4R*, the gene encoding the pancreatic polypeptide receptor Y4, which has been described as a satiety-stimulating receptor. We have investigated CNV of the *NPY4R* gene and analysed its relationship to BMI, waist circumference and self-reported dietary intake from 558 individuals (216 men and 342 women) representing a wide BMI range. The copy number for *NPY4R* ranged from 2 to 8 copies (average 4.6 ±0.8). Rather than the expected negative correlation, we observed a positive correlation between *NPY4R* copy number and BMI as well as waist circumference in women (Pearson's $r = 0.267$, $p = 2.65\times10^{-7}$ and $r = 0.256$, $p = 8\times10^{-7}$, respectively). Each additional copy of *NPY4R* correlated with 2.6 kg/m^2 increase in BMI and 5.67 cm increase in waist circumference ($p = 2.8\times10^{-5}$ and $p = 6.2\times10^{-5}$, respectively) for women. For men, there was no statistically significant correlation between CNV and BMI. Our results suggest that *NPY4R* genetic variation influences body weight in women, but the exact role of this receptor appears to be more complex than previously proposed.

OPEN ACCESS

Citation: Shebanits K, Andersson-Assarsson JC, Larsson I, Carlsson LMS, Feuk L, Larhammar D (2018) Copy number of pancreatic polypeptide receptor gene *NPY4R* correlates with body mass index and waist circumference. PLoS ONE 13(4): e0194668. https://doi.org/10.1371/journal.pone.0194668

Editor: Cheng Hu, Shanghai Diabetes Institute, CHINA

Received: November 23, 2017

Accepted: March 7, 2018

Published: April 5, 2018

Copyright: © 2018 Shebanits et al. This is an open access article distributed under the terms of the Creative Commons Attribution License, which permits unrestricted use, distribution, and reproduction in any medium, provided the original author and source are credited.

Data Availability Statement: The full dataset necessary for replication of the study findings is included in a Supporting Information file.

Funding: This study was supported by the grants from the Swedish Research Council (https://www.vr.se/) K2013-54X-11285-19 (LC) and K2013-55X-22189-01-2 (DL), the Swedish Brain Foundation (http://www.hjarnfonden.se/) FO2016-0217 (DL), and the Sahlgrenska University Hospital Regional Agreement on Medical Education and Research.

Introduction

Excessive weight gain has become one of the major health problems worldwide. According to a report from 2013, 37% of men and 38% of women had overweight or obesity [1]. Increased body mass index (BMI) is associated with increased mortality from cardiovascular disease, type 2-diabetes and several types of cancer [2]. Increased waist circumference (WC) is associated with abdominal obesity and increased risk for metabolic complications [3].

Obesity is a complex, polygenic, and highly heritable disease. Heritability of BMI ranges between 24–80% in family studies and 47–90% in twin studies (for review see[4]). Multiple studies have demonstrated that structural differences in the genome, such as copy number variation (CNV), are associated with variation in BMI [5] and obesity [6,7].

The funders had no role in study design, data collection and analysis, decision to publish, or preparation of the manuscript.

Competing interests: Prof. Carlsson reports grant from the Swedish Research Council and lecture fees from Johnson&Johnson, MSD and AstraZeneca, during the conduct of the study; Prof. Larhammar reports grants from Novo Nordisk, outside the submitted work, and the Swedish Research Council and the Swedish Brain Foundation (supporting this study). This does not alter our adherence to PLOS ONE policies on sharing data and materials.

A previous genetic screen of a cohort of obese German children carried out by the genetics company IntegraGen revealed an association between a genomic region on chromosome 10q11.22 and obesity (personal communication with Dr Jorg Hager). Among the genes located in the region, *NPY4R* was the strongest candidate for association with obesity and was found to have nonsynonymous single nucleotide polymorphisms (SNPs) that segregated with childhood obesity.

The copy number variable region on chromosome 10q11.22 spanning across the *NPY4R*, *SYT15* and *GPRIN2* genes has been previously described by several research groups [8–11]. The first study of CNV in this region and association with BMI reported an inverse correlation: a higher gene copy number was associated with reduced BMI in an elderly Chinese cohort [5]. A similar correlation was subsequently confirmed in a German cohort [6] and a Belgian cohort of children and adolescents with obesity and healthy adults with normal weight [12]. However, in a study of young Chinese individuals no CNV was detected in the 10q11.22 region [13].

The *NPY4R* gene encodes the Y4 receptor that responds to pancreatic polypeptide (PP). This gene is a strong candidate for body weight regulation because PP has been reported to be a potent appetite inhibitor [14]. There are four NPY-family receptors in humans and all of them are expressed in the brain, especially in the hypothalamic regions that are involved in the control of appetite and energy metabolism [15], as well as in the periphery (for Y4 see[16]).

PP is released from pancreatic PP-cells, previously called F cells, postprandially in proportion to caloric intake [17]. Intravenous administration of PP causes reduced energy intake in both individuals with normal weight and those with obesity [14,18]. Peripheral administration of PP decreases the hypothalamic expression of the potent hunger stimulants NPY, ghrelin and orexin, and increases anorexigenic urocortin in animal models [19].

PP affects appetite by acting through Y4 receptors in the regions that play a crucial role in energy balance, like the dorsal vagal complex, area postrema and the nucleus of the solitary tract in the brain stem [20,21], the arcuate nucleus [21], lateral hypothalamic area [22], and the paraventricular and ventromedial nuclei [21] of the hypothalamus.

To address the relationship between *NPY4R* copy number and obesity, we present here a study of 558 individuals with a wide range of BMIs. We investigated the associations between copy number and each of the following parameters: BMI, WC and self-reported dietary intake data from a validated questionnaire.

Materials and methods

Study populations

The study populations included participants from the Swedish Obese Subjects (SOS) study [23], the SOS reference study (SOS-ref) [24] and the SOS SibPair study [25]. The SOS study was started in 1987 and is a prospective, controlled, intervention study involving 4047 individuals; 2010 individuals have undergone bariatric surgery and 2037 conventional treatment (matched control group). Minimum BMI for inclusion was 38 kg/m^2 for women and 34 kg/m^2 for men. Average BMI at baseline was 42.2±4.5 kg/m^2 in the surgery group and 40.1±4.7 kg/m^2 in the control group. The SOS-ref study includes subjects from the Swedish cities Mölndal and Örebro. The subjects were randomly selected from a population registry to constitute a cross-sectional reference group to the SOS study [24]. The study includes 1135 subjects (46.5% men), average BMI is 25.2±3.8 kg/m^2. The SOS SibPair study [25] consists of 732 individuals from 154 Swedish nuclear families with sibling pairs discordant for obesity, defined as a BMI difference of at least 10 kg/m^2. BMI range was 16.9 to 57.8 kg/m^2. The SOS SibPair study was specifically designed to study genetic aspects of obesity.

First, 239 randomly chosen native Swedish individuals from the SOS reference study were investigated, and then 75 individuals from the SOS study and 244 individuals from the SOS SibPair cohort were added to increase the number of subjects with extreme BMI values. In total, 558 individuals from these three study populations were included in the present study (hereafter referred to as the study sample) with the aim to cover a wide BMI range (16.9-49.7 kg/m^2). Measurements of BMI and WC as well as self-reported dietary intake data on food and beverage intake were available for all individuals (See S1 Table).

All procedures performed in the study involving human participants were in accordance with the ethical standards of the local and regional review boards and with the 1964 Helsinki declaration and its later amendments or comparable ethical standards. Informed oral or written consent was obtained from all participants, according to the regional ethical review boards' guidelines and regulations. The following seven regional ethics review boards approved the study: Gothenburg, Lund (Region of Skåne), Linköping, Örebro, Karolinska Institute (Stockholm), Uppsala and Umeå.

Questionnaire data collection

All participants in SOS, SOS-ref and SOS SibPair completed a semi-quantitative dietary questionnaire on habitual food and beverage intake covering the last three months [26]. The subjects specified intake frequency of standard portions of different foods. The dietary questionnaire included 51 questions and had been validated against a 4-day food record [26,27] in groups of normal weight, overweight as well as adults with obesity. From the dietary questionnaire, total energy and macronutrient intake were calculated as well as energy intake from 8 carbohydrate- and / or fat-rich food groups (spreads, sandwiches, desserts, fruits, non-alcoholic beverages (excluding milk) salty snacks, candy, and buns and cakes).

Droplet digital PCR

We used droplet digital PCR (ddPCR) in order to study the copy number variation of the *NPY4R* gene (See S1 Table). Fluorescently labelled target (*NPY4R*) and reference (*RPPH1*) assays were designed according to the guidelines from Bio-Rad Laboratories.

The assay for *NPY4R* consists of the following primers and probe: forward primer: `5′- TG CATCCATTTGCATCG-3′`, reverse primer: `5′-CTGCAAGGCTTACTGTGACC-3′`, probe: `5′- TCAGCTGTTTGTTCCTGGGAGAA(FAM)-3′`. The assay for *RPPH1* consists of the following primers and probe: forward primer: `5′-CGCGCGAGGTCAGACT-3′`, reverse primer: `5′- GGTACCTCACCTCAGCCATT-3′`, Probe: `5′-(VIC)CCGGCGGATGCCTCCTT-3′`. Reference assay for replication was purchased from Bio-Rad Laboratories (*EIF2C1*, Cat#:100–31243).

DNA was digested with BstXI restriction enzyme (10U/μl, ThermoScientific, Cat#: ER1021) in Buffer 0 for 1 hour at 55˚C, followed by 20 min at 80˚C. A 20 μl mixture of 2×ddPCR Supermix for Probes (Bio-Rad, Cat#: 186–3010), forward and reverse primers for target and reference assay (final concentrations of 900nM each), probes for both assays (final concentrations of 250nM each) and 15 ng of digested DNA was emulsified with Bio-Rad Droplet Generator Oil (Bio-Rad, Cat#: 186–3005) in a Bio-Rad QX100^{TM} Droplet Generator (Bio-Rad, Cat#: 186–3001) according to the manufacturer's instructions. The droplets were then manually transferred to a 96-well plate (Eppendorf, Cat#: 951020362) and heat-sealed with Easy Pierce sealing foil sheets (Thermo Fisher Scientific, Cat#: AB-0757). Polymerase chain reaction was performed in a Bio-Rad C1000 thermal cycler (Bio-Rad, Cat#: 185–1197) with the following cycling parameters: 10 min at 95˚C (1 cycle), 30 s denaturation at 94˚C and 1 min annealing and extension at 58˚C (40 cycles), followed by 10 min at 98˚C and a hold at 12˚C. All steps had

a ramp rate of 2˚C/s. After the PCR, droplets were analysed using a Bio-Rad QX100 Droplet Reader (Bio-Rad, Cat#: 186–3001). Fluorescent data from each well were analysed with QuantaSoft software (v1.3.2), where copy number was calculated based on Poisson distribution[28]. We have tested the reliability of our copy number results by using the *EIF2C1* reference assay (which now is one of the Bio-Rad recommended reference assays for copy number analysis using ddPCR) on a randomly selected subset of samples, including samples with non-integer copy number.

Data analysis and statistics

For genotype frequency distribution, copy number data was binned to the closest integer (e.g. 2 = 1.5–2.49). First, Pearson correlation was used to assess whether there was a correlation between *NPY4R* copy number and BMI, WC, energy intake, energy intake adjusted to body weight, energy intake from different food groups and energy percent from macronutrients. Then, the effect of copy number change was estimated using linear mixed model. Any correlation within families for individuals from the SOS SibPair study were accounted for in the linear mixed model. Family ID was used as random effect, age and sex were included as covariates. The models were estimated using REML (Restricted Maximum Likelihood). All interpretations are similar to a normal linear regression and data is presented as an estimate and a standard error of estimate (SE). Non-normally distributed questionnaire data was analysed using Mann-Whitney U-test. False discovery rate analysis ($q<0.05$) was performed in order to correct for multiple testing. To account for known sex-related differences in BMI [29] and also to investigate any potential sex differences in the impact of CNV on body weight, we analysed our data for men and women together and separately.

Results

Basic characteristics of the study sample including age, weight, height, BMI and WC are summarized in Table 1.

Droplet digital PCR analysis demonstrated that *NPY4R* gene copy number varied from 2 to 8 (2.10–7.51 in men and 2.35–7.95 in women) with 4 being most common in BMI $\leq$25.0 kg/m^2 (average 4.26±0.61 for men and 4.09±0.62 for women). In the study group, 84% had 4 or 5 copies (average 4.71±0.80 for men and 4.53±0.81 for women) (Table 2).

In men and women combined, a positive correlation between *NPY4R* copy number and BMI was found (Pearson's r = 0.206, p = 4.85×10^{-7}). It was also found in women only (Pearson's r = 0.267, p = 2.65×10^{-7}) (Fig 1), whereas no statistically significant correlation between *NPY4R* copy number and BMI was found in men (Pearson's r = 0.098, p = 0.075). Linear

Table 1. Basic characteristics of the study sample.

Variables	Men N = 216		Women N = 342	
	Mean	Range	Mean	Range
Age (years)	50.8±11.1	18.1–78.6	48.4±9.4	22.2–72.4
Weight (kg)	95.5±20.0	53.0–179.0	88.3±22.0	43.7–146.6
Height (m)	1.79±0.06	1.56–2.00	1.66±0.06	1.48–1.83
BMI (kg/m^2)	29.7±5.5	17.9–49.7	32.1±7.8	16.9–49.6
Waist circumference (cm)	102.5±14.7	69.0–151.0	100.5±18.0	61.0–142.0
NPY4R copy number	4.58±0.80	2.10–7.51	4.53±0.81	2.35–7.95

Note: data presented as mean ± s.d.

https://doi.org/10.1371/journal.pone.0194668.t001

Table 2. CNV frequencies of *NPY4R*.

Binned copy number	Men N = 216	Women N = 342	BMI≤24.9 kg/m² N = 106	BMI 25.0–29.9 kg/m² N = 154	BMI≥30.0 kg/m² N = 297	Total N = 558
2	1	1	0	2	0	2
3	8	18	9	4	13	26
4	110	174	71	80	133	284
5	72	113	24	48	113	185
6	19	25	3	14	27	44
7	5	9	0	6	8	14
8	1	2	0	0	3	3

Note: data presented as mean ± s.d.

https://doi.org/10.1371/journal.pone.0194668.t002

mixed model analysis demonstrated that each additional copy corresponds to an increase of 2.60 kg/m^2 in BMI (SE = 0.50, p = 2.80x10^{-5}) in women. Waist circumference correlated with *NPY4R* copy number in men and women together (Pearson's r = 0.189, p = 3×10^{-6}) and in women only (Pearson's r = 0.256, p = 8×10^{-7}), but not in men (Pearson's r = 0.055, p = 0.21) (Fig 2). An increase of one copy was associated with 5.67 cm increase in WC (SE = 1.15, p = 16.02x10^{-5}) in women. We observed no correlation between *NPY4R* copy number and age, neither for the whole study group, nor for men and women separately.

Total energy intake, energy percent of macronutrients and energy intake from 8 food groups in relation to *NPY4R* copy number are presented in Table 3. No correlations were observed between total energy intake or energy intake from specific food groups and *NPY4R* copy number, neither in the entire study group, nor in men or women separately. No correlations were found between energy percent from carbohydrates, protein or fat and *NPY4R* copy number in the entire study group or in men or women separately. Total energy intake adjusted to body weight had a strong negative correlation with the *NPY4R* copy number in the whole study (Pearson's r = -0.199, p = 1×10^{-6}) sample and women separately (Pearson's r = -0.239, p = 4×10^{-6}). Each additional copy was associated with a decrease of 3.07 kcal/kg in the whole study sample (SE = 0.63, p = 4.78x10^{-6}) and a decrease of 3.49 kcal/kg in women separately

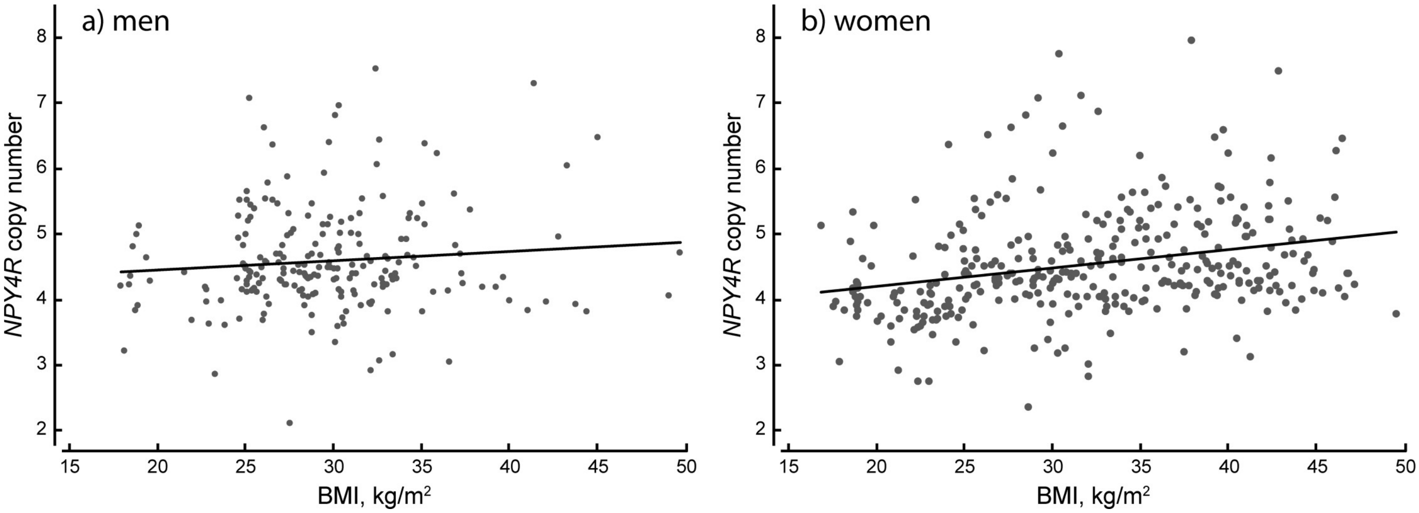

Fig 1. Correlation between BMI and *NPY4R* copy number. *NPY4R* copy number, determined by ddPCR, plotted against BMI in men (a) and women (b).

https://doi.org/10.1371/journal.pone.0194668.g001

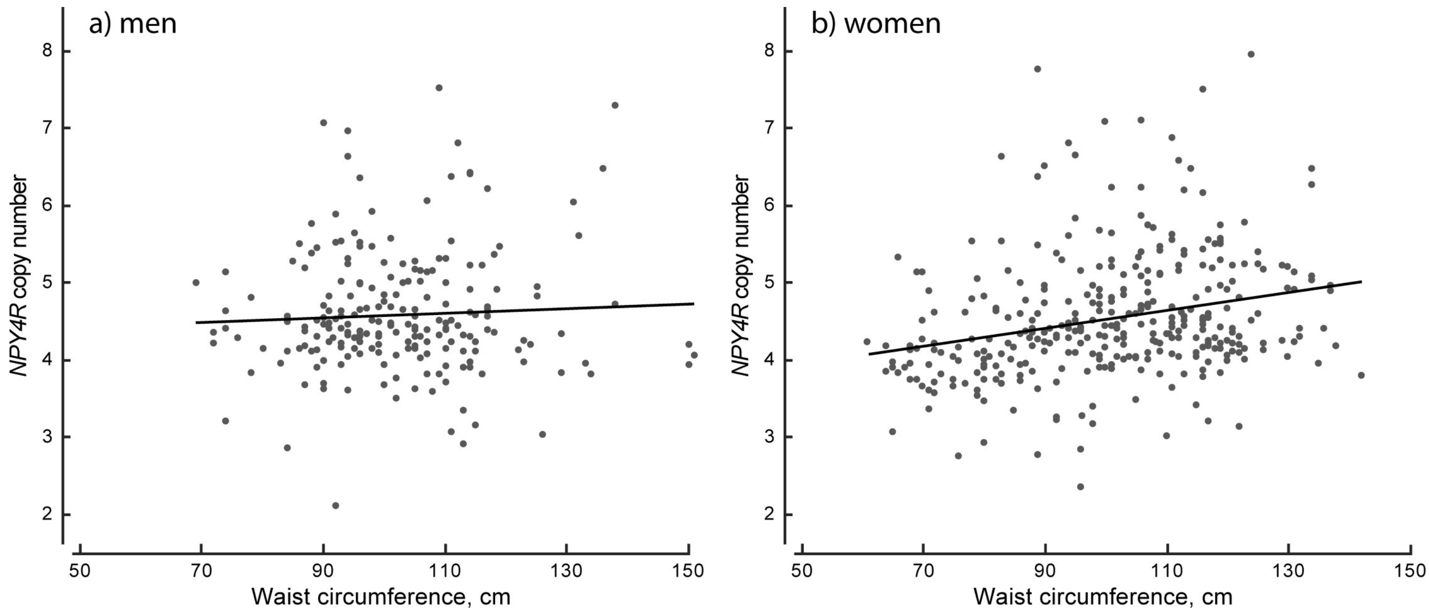

Fig 2. Correlation between waist circumference and *NPY4R* copy number. *NPY4R* copy number, determined by ddPCR, plotted against waist circumference in men (a) and women (b).

https://doi.org/10.1371/journal.pone.0194668.g002

(SE = 0.78, p = 1.28x10^{-4}). There was no statistically significant correlation between the total energy intake adjusted to body weight and the *NPY4R* copy number in men.

Discussion

NPY4R CNV in the study sample

Droplet digital PCR has recently emerged as the most accurate way for absolute DNA copy number quantification [30]. Here we report copy number state of the *NPY4R* gene in 558 adult

Table 3. Energy intake characteristics.

Variables	Men		Women	
	Mean	Range	Mean	Range
Total energy intake (kcal)	2932±1002	1240–7657	2349±791	1026–6338
Carbohydrates (g)	331±123	123–810	276±98	96–762
Protein (g)	112 ±38	47–247	94±31	31–223
Fat (g)	117±47	31–398	92±37	29–272
Energy per cent from carbohydrates	45±5	33–62	47±6	33–76
Energy per cent from proteins	16±2	10–21	16±2	7–23
Energy per cent from fat	35±4	18–47	35±5	16–48
Energy intake from spreads (kcal)	108±154	0–828	87±118	0–797
Energy intake from sandwiches (kcal)	666±352	71–2530	494±240	33–1624
Energy intake from desserts (kcal)	65±88	0–948	49±54	0–551
Energy intake from fruits (kcal)	133±140	0–1265	175±119	0–878
Energy intake from non-alcoholic beverages (kcal)	190±177	0–1076	125±137	0–976
Energy intake from snacks (kcal)	114±240	0–2975	80±145	0–1161
Energy intake from candy (kcal)	211±250	0–1756	188±240	0–2351
Energy intake from buns and cakes (kcal)	152±136	0–694	130±139	0–1496

Note: data presented as mean ± s.d.

https://doi.org/10.1371/journal.pone.0194668.t003

Swedish individuals from the SOS, SOS Ref and SOS SibPair cohorts, representing a wide range of BMIs. We investigated the relationship between the CNV and BMI, WC and self-reported energy intake.

We found that the copy number of *NPY4R* varies from 2 to 8 copies per genome. Our results demonstrate a positive correlation between *NPY4R* copy number and both BMI and WC for the entire study group and for women only. The findings we describe here differ from previous studies with respect to normal *NPY4R* copy number, the copy number distribution, and its association with body weight. Several of these studies reported that copy number loss in this genomic region was associated with weight gain. Such negative correlation has been described for an elderly Chinese cohort of 597 individuals [5], a German cohort of 3255 individuals [6] and a Belgian cohort of 622 individuals [12]. In contrast, a study of 12 females with Rett syndrome found a positive correlation of *NPY4R* copy number with weight gain [31]. A genome wide association study (GWAS) of obesity-related CNVs reported that three out of 430 individuals with obesity were "carrying this CNV", whereas none of the 379 controls with the normal weight did [10]. A study of 799 young Chinese individuals could not detect CNV of *NPY4R*, neither in subjects with obesity nor in subjects with normal weight [13].

Genetic differences between populations could be one of the reasons for differences between our results and the previous findings, since four of the previous studies have been performed in Asian cohorts [5,9,10,13]. However, our study of *NPY4R* copy number in a subset of samples from the 1000 Genomes Project (S2 Table) shows no striking copy number difference between samples from Asian and Caucasian populations. Thus, we suggest that these differences may be due to an incorrect assumption about the normal copy number of the *NPY4R* as well as methodological and cohort differences between our and earlier studies. Methodologically, most of the previous studies were based on SNP-arrays, aCGH and RT-PCR-based methods that require a reference copy number (most commonly set at 2 copies per genome) [5,9,13] or depend heavily on relative fluorescence data quality [32]. In contrast, we used the ddPCR method, which allows for more precise quantification of target nucleic acid [30] and that has been validated for absolute copy number determination [28,33]. It is equally [34] or more reliable than other molecular methods of copy number determination [35], depending on the copy number distribution and the complexity of the region. Incorrectly chosen reference copy number or inappropriate choice of reference gene in PCR-based copy number determination methods represent sources of errors in CNV-studies. Therefore, we have used two reference assays: *RPPH1* and *EIF2C1*. We initially chose *RPPH1* as a reference gene because it is a well-known single-copy gene [36]. *EIF2C1*, a newer and now recommended reference assay for CNV detection, was used as a control in a subset of the samples. We found no differences in the results obtained with *RPPH1* compared with *EIF2C1*.

Selection of study populations is an important factor in obesity studies. Our study population consists of Swedish adults of both sexes and covers a wide range of BMIs (for details see Table 1). When studying BMI, it is important to take into consideration both the sex and age distribution. Age differences were accounted for using age as a covariate in the linear regression analyses. To account for sex-related differences in BMI, we analysed men and women together as well as separately.

Inverse correlation between body weight, waist circumference and *NPY4R* copy number

In our study sample, both BMI and WC exhibited positive correlation with the gene copy number. Our results in women demonstrated that for each additional copy of *NPY4R* the BMI increased with 2.60 kg/m^2. In comparison, in men and women each *FTO* risk-allele increases

BMI by 0.34–0.46 kg/m^2 [37]. The analyses of the correlations in the separate sexes showed a correlation in women but not in men. Studies indicate that there are sex-specific genetic factors contributing to obesity development [29]. Our finding of a correlation between *NPY4R* copy number and both BMI and WC in women, but not in men, adds support to the idea that different genes contribute to the variation in BMI in women and men.

We addressed the question whether dietary intake correlates with *NPY4R* copy number by analysing self-reported food intake. We observed no correlation between *NPY4R* copy number and total energy intake or energy percent of macronutrients, which may indicate that *NPY4R* influences body weight through metabolic pathways, rather than food intake. Alternatively, the lack of associations between *NPY4R* copy number and dietary intake may be due to the large variation in daily energy need in our study sample that consists of individuals with BMI ranging from 17–50 kg/m^2. There is also a well-known misreporting of self-reported food intake data affecting large population-based samples [38] as well as sub-groups of different BMIs [39,40]. Under reporters of energy intake are more often obese and overestimation of energy intake are more often seen in normal weight groups [38]. Also depending on BMI, misreporting of energy intake can be specific so that social desirable foods (i.e. high-fat, sugar-rich foods and beverages) are more often underreported by subjects with obesity and low-fat, fibre-rich foods are more often over-reported by normal weight and underweight groups [38,41]. The present study includes subjects covering a large BMI-interval. The questionnaire used in the present study has been found to be equally valid and reproducible for individuals with obesity as well as individuals with normal weight [26] and has previously been used in normal weight, overweight and obese study groups [23,40,42].

We have investigated the relationship between total energy intake adjusted to body weight and *NPY4R* copy number. We found a negative correlation for the whole study sample and women separately. Taking into account the role of Y4 and PP in mediation of satiety [14,18], such negative correlation would mean that individuals with more *NPY4R* copies experience more satiety. At the same time, we see that individuals with more *NPY4R* copies have higher BMI and WC. Such seemingly contradictory findings might be explained by differences in energy metabolism between individuals with low and high *NPY4R* copy number. It is also important to mention that the role of PP (and hence, its receptor Y4) in food intake and energy metabolism regulation is no so univocal. In a long-term study of hormonal levels after weight loss in adults [43], the level of satiety hormones was significantly decreased while the levels of hunger hormones increased significantly, as if trying to bring the individuals back to higher body weight. Surprisingly, the level of PP increased after weight loss as if it were a hunger-inducing signal. The same effect was observed in a study of weight loss in obese children [44]. In the light of aforementioned possible misreporting in self-reported food/energy intake and the complex role of the PP/Y4 system we think that these results should be interpreted with great care.

We observed that *NPY4R* copy number is not always an integer. Therefore, we binned the data to whole numbers (±0.5) for genotype frequency estimation. Variation in the data due to ddPCR methodology is unlikely given the precision of the method [28] and consistency of non-integer results despite troubleshooting [45] and replication. We instead suggest that non-integer copy number may be a result of somatic mosaicism in peripheral blood DNA. Previously reported analysis of somatic mosaicism in tissues of healthy donors have revealed CNV of multiple regions, some spanning genes [46]. Also, mosaicism of inversion polymorphisms has been demonstrated in peripheral blood [47]. The investigated rearrangements were more abundant in adults than in new-borns, suggesting gradual accumulation of frequent postnatal rearrangements [47].

Conclusions

We found a positive correlation between *NPY4R* copy number and BMI and WC in women only, suggesting that the role of *NPY4R* in body weight may be more complex than previously thought. Thus, our results also lend support to the belief that there are different genetic contributors to BMI variation in men and women.

Our findings of a positive *NPY4R* copy number correlation with BMI is opposite to what we expected, based on previous studies of *NPY4R* and its ligand PP. This invites further investigation of their specific roles in appetite regulation and energy metabolism.

Supporting information

S1 Table. Phenotype and genotype data. Full phenotype and *NPY4R* copy number data for 558 individuals used in this study.
(DOCX)

S2 Table. *NPY4R* copy number determined by ddPCR in Chinese and Caucasian samples from 1000 Genomes Project.
(DOCX)

Acknowledgments

We would like to acknowledge all the participants of our study for making this work possible. Our study was funded by the Swedish Research Council, the Swedish Brain Foundation, and the Sahlgrenska University Hospital Regional Agreement on Medical Education and Research. We thank Leif Andersson for access to the droplet digital PCR facility and Jennifer Meadows for input and guidance during the initial setup of the method.

Author Contributions

Conceptualization: Kateryna Shebanits, Dan Larhammar.

Data curation: Johanna C. Andersson-Assarsson.

Formal analysis: Kateryna Shebanits.

Funding acquisition: Lena M. S. Carlsson, Dan Larhammar.

Investigation: Kateryna Shebanits.

Resources: Johanna C. Andersson-Assarsson, Lena M. S. Carlsson.

Supervision: Dan Larhammar.

Writing – original draft: Kateryna Shebanits.

Writing – review & editing: Johanna C. Andersson-Assarsson, Ingrid Larsson, Lena M. S. Carlsson, Lars Feuk, Dan Larhammar.

References

1. Ng M, Fleming T, Robinson M, Thomson B, Graetz N, Margono C, et al. Global, regional, and national prevalence of overweight and obesity in children and adults during 1980–2013: A systematic analysis for the Global Burden of Disease Study 2013. Lancet. 2014; 384: 766–781. https://doi.org/10.1016/S0140-6736(14)60460-8 PMID: 24880830
2. Prospective Studies Collaboration. Body-mass index and cause-specific mortality in 900 000 adults: collaborative analyses of 57 prospective studies. Lancet. Elsevier Ltd; 2009; 373: 1083–1096. https://doi.org/10.1016/S0140-6736(09)60318-4 PMID: 19299006

3. Aronne LJ, Segal KR. Adiposity and fat distribution outcome measures: assessment and clinical implications. Obes Res. 2002; 10 Suppl 1: 14S–21S. https://doi.org/10.1038/oby.2002.184 PMID: 12446853

4. Elks CE, Hoed M Den, Zhao JH, Sharp SJ, Wareham NJ, Loos RJF, et al. Variability in the heritability of body mass index: A systematic review and meta-regression. Front Endocrinol (Lausanne). 2012; 3: 1–16. https://doi.org/10.3389/fendo.2012.00029 PMID: 22645519

5. Sha B-Y, Yang T-L, Zhao L-J, Chen X-D, Guo Y, Chen Y, et al. Genome-wide association study suggested copy number variation may be associated with body mass index in the Chinese population. J Hum Genet. Nature Publishing Group; 2009; 54: 199–202. https://doi.org/10.1038/jhg.2009.10 PMID: 19229253

6. Jarick I, Vogel CIG, Scherag S, Schäfer H, Hebebrand J, Hinney A, et al. Novel common copy number variation for early onset extreme obesity on chromosome 11q11 identified by a genome-wide analysis. Hum Mol Genet. 2011; 20: 840–52. https://doi.org/10.1093/hmg/ddq518 PMID: 21131291

7. Falchi M, El-Sayed Moustafa JS, Takousis P, Pesce F, Bonnefond A, Andersson-Assarsson JC, et al. Low copy number of the salivary amylase gene predisposes to obesity. Nat Genet. Nature Publishing Group; 2014; 46: 492–7. https://doi.org/10.1038/ng.2939 PMID: 24686848

8. Sebat J, Lakshmi B, Troge J, Alexander J, Young J, Lundin P, et al. Large-scale copy number polymorphism in the human genome. Science. 2004; 305: 525–8. https://doi.org/10.1126/science.1098918 PMID: 15273396

9. Park H, Kim J-I, Ju YS, Gokcumen O, Mills RE, Kim S, et al. Discovery of common Asian copy number variants using integrated high-resolution array CGH and massively parallel DNA sequencing. Nat Genet. 2010; 42: 400–5. https://doi.org/10.1038/ng.555 PMID: 20364138

10. Wang K, Li W-D, Glessner JT, Grant SFA, Hakonarson H, Price RA. Large copy-number variations are enriched in cases with moderate to extreme obesity. Diabetes. 2010; 59: 2690–4. https://doi.org/10.2337/db10-0192 PMID: 20622171

11. Sudmant PH, Kitzman JO, Antonacci F, Alkan C, Malig M, Tsalenko A, et al. Diversity of human copy number variation and multicopy genes. Science. 2010; 330: 641–6. https://doi.org/10.1126/science.1197005 PMID: 21030649

12. Aerts E, Beckers S, Zegers D, Van Hoorenbeeck K, Massa G, Verrijken A, et al. CNV analysis and mutation screening indicate an important role for the NPY4R gene in human obesity. Obesity. 2016; 24: 970–976. https://doi.org/10.1002/oby.21435 PMID: 26921218

13. Sun C, Cao M, Shi J, Li L, Miao L, Hong J, et al. Copy number variations of obesity relevant loci associated with body mass index in young Chinese. Gene. Elsevier B.V.; 2013; 516: 198–203. https://doi.org/10.1016/j.gene.2012.12.081 PMID: 23291416

14. Jesudason DR, Monteiro MP, McGowan BMC, Neary NM, Park AJ, Philippou E, et al. Low-dose pancreatic polypeptide inhibits food intake in man. Br J Nutr. 2007; 97: 426–9. https://doi.org/10.1017/S0007114507336799 PMID: 17313701

15. Fetissov SO, Kopp J, Hökfelt T. Distribution of NPY receptors in the hypothalamus. Neuropeptides. 2004; 38: 175–188. https://doi.org/10.1016/j.npep.2004.05.009 PMID: 15337370

16. Lundell I, Blomqvist AG, Berglund MM, Schober DA, Johnson D, Statnick MA, et al. Cloning of a human receptor of the NPY receptor family with high affinity for pancreatic polypeptide and peptide YY. J Biol Chem. 1995; 270: 29123–8. https://doi.org/10.1074/jbc.270.49.29123 PMID: 7493937

17. Huda MSB, Wilding JPH, Pinkney JH. Gut peptides and the regulation of appetite. Obes Rev. 2006; 7: 163–182. https://doi.org/10.1111/j.1467-789X.2006.00245.x PMID: 16629873

18. Batterham RL. Pancreatic Polypeptide Reduces Appetite and Food Intake in Humans. J Clin Endocrinol Metab. 2003; 88: 3989–3992. https://doi.org/10.1210/jc.2003-030630 PMID: 12915697

19. Asakawa A, Inui A, Yuzuriha H, Ueno N, Katsuura G, Fujimiya M, et al. Characterization of the effects of pancreatic polypeptide in the regulation of energy balance. Gastroenterology. 2003; 124: 1325–1336. https://doi.org/10.1016/S0016-5085(03)00216-6 PMID: 12730873

20. Kojima S, Ueno N, Asakawa A, Sagiyama K, Naruo T, Mizuno S, et al. A role for pancreatic polypeptide in feeding and body weight regulation. Peptides. 2007; 28: 459–63. https://doi.org/10.1016/j.peptides.2006.09.024 PMID: 17207558

21. Lin S, Shi Y-C, Yulyaningsih E, Aljanova A, Zhang L, Macia L, et al. Critical role of arcuate Y4 receptors and the melanocortin system in pancreatic polypeptide-induced reduction in food intake in mice. PLoS One. 2009; 4: e8488. https://doi.org/10.1371/journal.pone.0008488 PMID: 20041129

22. Campbell RE, Smith MS, Allen SE, Grayson BE, Ffrench-Mullen JMH, Grove KL. Orexin neurons express a functional pancreatic polypeptide Y4 receptor. J Neurosci. 2003; 23: 1487–97. Available: http://www.ncbi.nlm.nih.gov/pubmed/12598637 PMID: 12598637

23. Sjöström L, Lindroos A-K, Peltonen M, Torgerson J, Bouchard C, Carlsson B, et al. Lifestyle, diabetes, and cardiovascular risk factors 10 years after bariatric surgery. N Engl J Med. 2004; 351: 2683–2693. https://doi.org/10.1056/NEJMoa035622 PMID: 15616203
24. Larsson I, Bertéus Forslund H, Lindroos AK, Lissner L, Näslund I, Peltonen M, et al. Body composition in the SOS (Swedish Obese Subjects) reference study. Int J Obes Relat Metab Disord. 2004; 28: 1317–1324. https://doi.org/10.1038/sj.ijo.0802732 PMID: 15314632
25. Carlsson LMS, Jacobson P, Walley A, Froguel P, Sjöström L, Svensson PA, et al. ALK7 expression is specific for adipose tissue, reduced in obesity and correlates to factors implicated in metabolic disease. Biochem Biophys Res Commun. Elsevier Inc.; 2009; 382: 309–314. https://doi.org/10.1016/j.bbrc.2009.03.014 PMID: 19275893
26. Lindroos AK, Lissner L, Sjöström L. Validity and reproducibility of a self-administered dietary questionnaire in obese and non-obese subjects. Eur J Clin Nutr. 1993; 47: 461–481. PMID: 8404782
27. Lissner L, Lindroos AK, Sjöström L. Swedish obese subjects (SOS): an obesity intervention study with a nutritional perspective. Eur J Clin Nutr. 1998; 52: 316–22. https://doi.org/10.1038/sj.ejcn.1600567 PMID: 9630380
28. Pinheiro LB, Coleman VA, Hindson CM, Herrmann J, Hindson BJ, Bhat S, et al. Evaluation of a droplet digital polymerase chain reaction format for DNA copy number quantification. Anal Chem. 2012; 84: 1003–1011. https://doi.org/10.1021/ac202578x PMID: 22122760
29. Schousboe K, Willemsen G, Kyvik KO, Mortensen J, Boomsma DI, Cornes BK, et al. Sex differences in heritability of BMI: a comparative study of results from twin studies in eight countries. Twin Res. 2003; 6: 409–421. https://doi.org/10.1375/136905203770326411 PMID: 14624725
30. Hindson BJ, Ness KD, Masquelier DA, Belgrader P, Heredia NJ, Makarewicz AJ, et al. High-throughput droplet digital PCR system for absolute quantitation of DNA copy number. Anal Chem. 2011; 83: 8604–8610. https://doi.org/10.1021/ac202028g PMID: 22035192
31. Artuso R, Papa FT, Grillo E, Mucciolo M, Yasui DH, Dunaway KW, et al. Investigation of modifier genes within copy number variations in Rett syndrome. J Hum Genet. 2011; 56: 508–15. https://doi.org/10.1038/jhg.2011.50 PMID: 21593744
32. Chu JH, Rogers A, Ionita-Laza I, Darvishi K, Mills RE, Lee C, et al. Copy number variation genotyping using family information. BMC Bioinformatics. 2013; 14: 157. https://doi.org/10.1186/1471-2105-14-157 PMID: 23656838
33. Tayoun ANA, Mason-Suares H, Frisella AL, Bowser M, Duffy E, Mahanta L, et al. Targeted Droplet-Digital PCR as a Tool for Novel Deletion Discovery at the DFNB1 Locus. Hum Mutat. 2016; 37: 119–26. https://doi.org/10.1002/humu.22912 PMID: 26444186
34. Svobodová I, Pazourková E, Hořínek A, Novotná M, Calda P, Korabečná M. Performance of Droplet Digital PCR in Non-Invasive Fetal RHD Genotyping—Comparison with a Routine Real-Time PCR Based Approach. PLoS One. 2015; 10: e0142572. https://doi.org/10.1371/journal.pone.0142572 PMID: 26562517
35. Sillence KA, Roberts LA, Hollands HJ, Thompson HP, Kiernan M, Madgett TE, et al. Fetal sex and RHD genotyping with digital PCR demonstrates greater sensitivity than real-time PCR. Clin Chem. 2015; 61: 1399–1407. https://doi.org/10.1373/clinchem.2015.239137 PMID: 26354802
36. Baer M, Nilsen TW, Costigan C, Altman S. Structure and transcription of a human gene for H1 RNA, the RNA component of human RNase P. Nucleic Acids Res. 1990; 18: 97–103. Available: http://www.pubmedcentral.nih.gov/articlerender.fcgi?artid=330208&tool=pmcentrez&rendertype=abstract PMID: 2308839
37. Frayling TM, Timpson NJ, Weedon MN, Zeggini E, Freathy RM, Lindgren CM, et al. A common variant in the FTO gene is associated with body mass index and predisposes to childhood and adult obesity. Science. 2007; 316: 889–94. https://doi.org/10.1126/science.1141634 PMID: 17434869
38. Johansson L, Solvoll K, Bjørneboe GE, Drevon CA. Under- and overreporting of energy intake related to weight status and lifestyle in a nationwide sample. Am J Clin Nutr. 1998; 68: 266–74. https://doi.org/10.1152/japplphysiol.00110.2005 PMID: 9701182
39. Macdiarmid J, Blundell J. Assessing dietary intake: Who, what and why of under-reporting. Nutr Res Rev. 1998; 11: 231–53. https://doi.org/10.1079/NRR19980017 PMID: 19094249
40. Heitmann BL, Lissner L. Dietary underreporting by obese individuals—is it specific or non-specific? BMJ. 1995; 311: 986–9. PMID: 7580640
41. Lissner L. Measuring food intake in studies of obesity. Public Health Nutr. 2002; 5: 889–892. https://doi.org/10.1079/PHN2002388 PMID: 12638596
42. Torgerson JS, Hauptman J, Boldrin MN, Sjöström L. XENical in the prevention of diabetes in obese subjects (XENDOS) study: a randomized study of orlistat as an adjunct to lifestyle changes for the

prevention of type 2 diabetes in obese patients. Diabetes Care. 2004; 27: 155–61. https://doi.org/10.4158/EP.12.S1.31 PMID: 14693982

43. Sumithran P, Prendergast L. Long-term persistence of hormonal adaptations to weight loss. Engl J. 2011; 365: 1597–604. https://doi.org/10.1056/NEJMoa1105816 PMID: 22029981

44. Reinehr T, Enriori PJ, Harz K, Cowley MA, Roth CL. Pancreatic polypeptide in obese children before and after weight loss. Int J Obes (Lond). 2006; 30: 1476–81. https://doi.org/10.1038/sj.ijo.0803393 PMID: 16702998

45. Mazaika E, Homsy J. Digital Droplet PCR: CNV Analysis and Other Applications. Curr Protoc Hum Genet. 2014; 82: 7.24.1–7.24.13. https://doi.org/10.1002/0471142905.hg0724s82 PMID: 25042719

46. Piotrowski A, Bruder CEG, Andersson R, De Ståhl TD, Menzel U, Sandgren J, et al. Somatic mosaicism for copy number variation in differentiated human tissues. Hum Mutat. 2008; 29: 1118–1124. https://doi.org/10.1002/humu.20815 PMID: 18570184

47. Flores M, Morales L, Gonzaga-Jauregui C, Domínguez-Vidaña R, Zepeda C, Yañez O, et al. Recurrent DNA inversion rearrangements in the human genome. Proc Natl Acad Sci U S A. 2007; 104: 6099–6106. https://doi.org/10.1073/pnas.0701631104 PMID: 17389356
